# The Role of Neurodevelopmental Pathways in Brain Tumors

**DOI:** 10.3389/fcell.2021.659055

**Published:** 2021-04-27

**Authors:** Rachel N. Curry, Stacey M. Glasgow

**Affiliations:** ^1^Department of Neuroscience, Baylor College of Medicine, Center for Cell and Gene Therapy, Houston, TX, United States; ^2^Integrative Molecular and Biomedical Sciences, Graduate School of Biomedical Sciences, Baylor College of Medicine, Houston, TX, United States; ^3^Neurobiology Section, Division of Biological Sciences, University of California, San Diego, San Diego, CA, United States; ^4^Neurosciences Graduate Program, University of California, San Diego, San Diego, CA, United States; ^5^Biomedical Sciences Graduate Program, University of California, San Diego, San Diego, CA, United States

**Keywords:** neurodevelopment, brain tumors, glioma, transcription factor, signaling pathway

## Abstract

Disruptions to developmental cell signaling pathways and transcriptional cascades have been implicated in tumor initiation, maintenance and progression. Resurgence of aberrant neurodevelopmental programs in the context of brain tumors highlights the numerous parallels that exist between developmental and oncologic mechanisms. A deeper understanding of how dysregulated developmental factors contribute to brain tumor oncogenesis and disease progression will help to identify potential therapeutic targets for these malignancies. In this review, we summarize the current literature concerning developmental signaling cascades and neurodevelopmentally-regulated transcriptional programs. We also examine their respective contributions towards tumor initiation, maintenance, and progression in both pediatric and adult brain tumors and highlight relevant differentiation therapies and putative candidates for prospective treatments.

## Introduction

**FIGURE 1:**
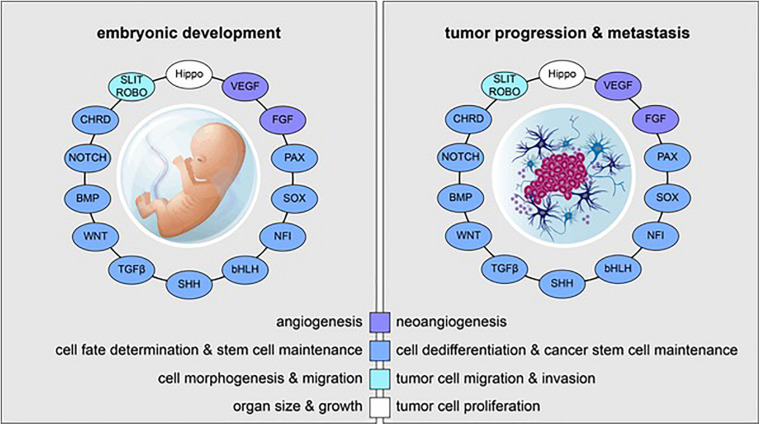
Reactivation of embryonic and developmental signaling pathways in cancer. Examples of embryonic signaling pathways, transcription factor families, and their respective roles in embryonic development **(left)** and tumor progression and metastasis **(right)** are shown. Signaling pathways and transcriptional cascades that are essential for proper in utero development are often reactivated in the context of cancer.

Despite extraordinary technological, pharmacological and immunotherapeutic advances in other cancer fields, overall survival for malignant central nervous system (CNS) tumors remains at a dismal 36% with the most aggressive subtypes conferring a survival rate of merely 5% (Ostrom et al., 2016). CNS tumors encompass a broad group of brain and spinal neoplasms, which include astrocytic oligodendroglial, ependymal, choroid plexus, neuronal, and mixed neuronal-glial subtypes as well as tumors of the pineal region and tumors of the spinal and paraspinal nerves (Figure 1). Within these groups, glioblastoma (GBM) stands out as the most lethal variant affecting the adult population and represents 52% of all primary brain tumors (Ostrom et al., 2016). Malignant gliomas also represent 53% of tumors occurring in children less than 14 years of age, with the most common variant (17.6%) presenting as pilocytic astrocytoma. Embryonal tumors account for an additional 15% of all primary brain tumors in children of this age group, 61.7% of which are medulloblastomas. Collectively, brain and other CNS tumors are the most common cancer in children and account for the 10th leading cause of death for adult men and women in the United States (Ostrom et al., 2016).

Devastating survival rates for the more lethal brain tumor variants are in large part due to poorly defined tumor margins caused by rampant proliferation and unchecked promigratory pathways. These diffuse tumor boundaries have proven impossible to eradicate using current therapeutic techniques (Jermyn et al., 2016) and thus, give rise to disease recurrence as well as the widespread and ultimately fatal dissemination of tumor cells throughout the brain. Surgical treatment protocols rely on complete neurosurgical resection as well as radiation therapy and chemotherapy but fail to eliminate the highly-disseminated infiltrates associated with the diffuse nature of these tumors (Juratli et al., 2013). Alternative, non-invasive treatments using systemic chemotherapeutics have been limited by the existence of the blood-brain barrier; despite efforts to circumvent this barrier using convection-enhanced delivery, drug-impregnated intratumoral wafers or intrathecal injections, effective drug delivery that targets individual tumor cells and spares the surrounding neuropil has yet to be achieved (Peiris et al., 2015). Notwithstanding these efforts to produce novel therapeutic strategies for malignant brain tumors, standard treatment regimens have remained unchanged for over 30 years.

The robust body of scientific literature confirming roles for developmental genes in oncogenesis, tumor invasiveness and tumor metastasis has resulted in a novel subclass of oncogenes termed fetal oncogenes, which demonstrate high expression in both embryonic development and cancer but minimal expression following the postnatal period. The existence of fetal oncogenes emphasizes the myriad parallels that exist between human *in utero* development and tumorigenesis (Slamon and Cline, 1984; Baker et al., 2016). Marginal expression of fetal oncogenes in non-tumor tissue can allow for efficacious targeting of cancer cells with minimal unwanted and potentially lethal off-target effects, thus rendering these tumor-specific antigens exploitable tumor attributes that can be used for immunotherapeutic gain. The resurgence of embryonic pathways in cancer has thus highlighted the relevance of developmental paradigms in oncologic diseases and emphasizes the importance of developmental biology in achieving novel therapeutic strategies for malignant glioma. In this review, we will explore cancers of the brain from the perspective of developmental biology. We will focus on defining critical roles for neurodevelopmental signaling events and lineage-specific transcription factors (TFs) and their respective contributions toward the hallmark features of oncogenesis, tumor maintenance and tumor evolutions. Specifically, we will discuss how these developmental mechanisms regulate stem cell identity, cellular proliferation, and cell differentiation. We will conclude our discussion with an examination of how these developmental mechanisms might be further leveraged to uncover new therapeutic approaches.

## Molecular Profiles of Brain Tumors

The litany of genetic abnormalities attributed to neurological cancers is extensive and includes a list of molecular and signaling cascades that partake in the development and sustenance of organ systems outside of the CNS. Altered expression profiles of epidermal growth factor (EGF; Di Carlo et al., 1992), platelet-derived growth factor (PDGF; Smits and Funa, 1998), and vascular endothelial growth factor (VEGF; Machein and Plate, 2000) and their respective receptors have each been implicated in the development and progression of primary brain tumors. Gross chromosomal anomalies in 1p19q have also been reported in glioma subsets (Reitman and Yan, 2010; Wu et al., 2010). Hypermethylation of O*6*-methylguanine-DNA methyltransferase (MGMT) has been implicated as a prognostic marker for a number of neurological tumor subsets and is believed to play an important role in the resistance of neurological tumors to therapeutic alkylating agents such as temozolomide (TMZ) and nitrosourea derivatives (Mellai et al., 2012). Mutations in isocitrate dehydrogenase (IDH) 1/2 enzymes have been identified as major genetic drivers of diffuse gliomas (Balss et al., 2008; Parsons et al., 2008; Ichimura et al., 2009; Watanabe et al., 2009). The most common mutations occurring in GBM remain tumor protein 53 (p53), phosphatase and tensin homolog (PTEN) and neurofibromin 1 (NF1), which account for ∼70% percent of the mutational burden in malignant glioma (Cerami et al., 2012; Gao et al., 2013; Zhang M. et al., 2019). In addition, recent research has identified a number of other irregularly expressed or genetically altered molecular targets in these cancers, including a variety of neurodevelopmental factors which will be reviewed here (Table 1).

**TABLE 1:** Genetic alterations occurring in pediatric and adult brain tumors.

**Adult CNS Tumors**			

**Oligodendroglioma**	**Astrocytoma**	**Glioblastoma**	
1p/19q co-deletion	ATRX	IDH1/2 WT or mutant	
CIC	BRAF FUSION	EGFR (EGFRviii)	
FUBP1	CDKN2A/B	MGMT promoter hypermethylation	
IDH1/2 WT or mutant	EGFR (EGFRviii)	PTEN	
TERT promoter mutation	IDH1/2 mutant p53	RB TERT promoter mutation	

**Pediatric tumors**			

**Ependymoma**	**Medulloblastoma**	**Low grade glioma**	**High grade glioma**

22q loss	APC	BRAF FUSION	ACVR1
NF2	BRAC2	BRAF V600E	FGFR1
RELA-fusion	CTNNB1	FGFR1	H3.3 K27
YAP1-fusion	Isochromosome	MYB	H3.1 K27
	17q	NF1	H3.3 G34R
	Monosomy 6	TSC1/2	
	NMYC		
	PTCH1		
	SMARCB1		
	SMARCA4		
	SMO		
	SUFU		
	TP53		

The discovery of IDH1/2 mutations in glioma has fueled the classification of malignant gliomas at the phenotypic, molecular, and genetic levels, ultimately leading to the definition of three major glioma subtypes – mesenchymal, classical, and proneural (Phillips et al., 2006; Verhaak et al., 2010; Louis et al., 2016). Subsequent studies have defined several molecularly distinct groups and determined a high level of intratumoral and intertumoral heterogeneity. This variability has confounded treatment and is a contributing factor to the high incidence of recurrence for many of these tumors. The development of single cell RNA sequencing (scRNAseq) methodologies has opened the door to uncovering the multitude of cellular profiles present within these tumors and has to helped define discrete cell populations and their associated molecular programs. Using single cell genomics in parallel with other transcriptomic techniques has allowed scientists a more detailed look into tumor genetic heterogeneity, tumor epigenetics and spatial contributions to tumorigenesis. Most recently, these approaches have revealed categorically distinct cell types and cellular states, which can be used to describe lineage hierarchy within tumors.

The similarities between developmental cell types, tumor expression profiles, and cancer cellular hierarchies have reshaped the way in which oncological research views cellular differentiation within tumors and have begun to shed light on tumor cells of origin. Cellular differentiation and cells of origin have perhaps been best studied in malignant gliomas and represent some of the most well-characterized cells in CNS tumor biology. Based on multimodal analyses, intratumoral heterogeneity in malignant glioma has been predominantly characterized by cell cycle disturbances and further correlated with similarity to distinct neural cell populations (Tirosh et al., 2016; Venteicher et al., 2017; Filbin et al., 2018; Neftel et al., 2019). In glioma, these neurodevelopmental cell types are reminiscent of neural progenitors and differentiated glial cell lineages, including astrocytes and oligodendrocytes. In pediatric and adult GBMs, cellular heterogeneity is driven by four distinct cellular states that are linked to genetic alterations: neural-progenitor like [cyclin dependent kinase 4 (CDK4) amplification], oligodendrocyte-progenitor like (PDGFR amplification), astrocyte-progenitor like (EGFR amplification), and mesenchymal-progenitor like (NF1 amplification). While GBMs tend to show enrichment for a particular state, cells from each group can be identified intratumorally (Neftel et al., 2019). Similar cancer cell hierarchies have been described for high-grade pediatric gliomas, as well as for IDH mutant and IDH wildtype adult gliomas (Tirosh et al., 2016; Venteicher et al., 2017; Filbin et al., 2018; Neftel et al., 2019; Couturier et al., 2020). With respect to the latter, scRNAseq strategies have revealed a tri-lineage cancer hierarchy uniformly deriving from a glial progenitor-like cell with notable similarities in stemness to what has been termed a glioma stem cell (GSC). In these tumors, progenitor cells are the most rapidly dividing cells and demonstrate the most potent tumorigenic properties, lending support to the notion that malignant gliomas develop along a neurodevelopmental trajectory (Couturier et al., 2020).

Other scRNAseq studies have examined developmental cellular diversity amongst a number of CNS tumor subtypes. Whereas previous bulk tumor profiling of medulloblastomas had defined four transcriptomically distinct subgroups of cells within the tumor, including sonic hedgehog (SHH)-activated, wingless-type MMTV integration site family (WNT)-activated, group 3, and group 4 (Thompson et al., 2006; Kool et al., 2008; Northcott et al., 2011), more recent single cell analyses have identified three predominate cell populations. These studies have pointed to distinct groups of cycling cells, undifferentiated cells, and cells on a neuronal differentiation trajectory as major drivers of clinical outcomes (Hovestadt et al., 2019). Likewise, pediatric ependymomas have been demonstrated to contain a multitude of cellular constituents that closely resemble the transcriptional programs of normal brain development. The developmental profiles of these cancer cells show striking similarity to the transcriptomic signatures of a number of non-tumor neural cell types. These signatures present in a number of developmentally differentiated states ranging from undifferentiated cells to neuronal and glial subpopulations whose aberrant developmental trajectories could be driving pathogenesis (Gojo et al., 2020). Interestingly, pediatric tumors appear to have the highest proportion of cycling or undifferentiated cells that parallels the rapid expansion and proliferation of certain neural subsets known to occur throughout the perinatal and adolescent stages of development (Filbin et al., 2018; Neftel et al., 2019).

## Stem Cells in Brain Tumors

**FIGURE 2:**
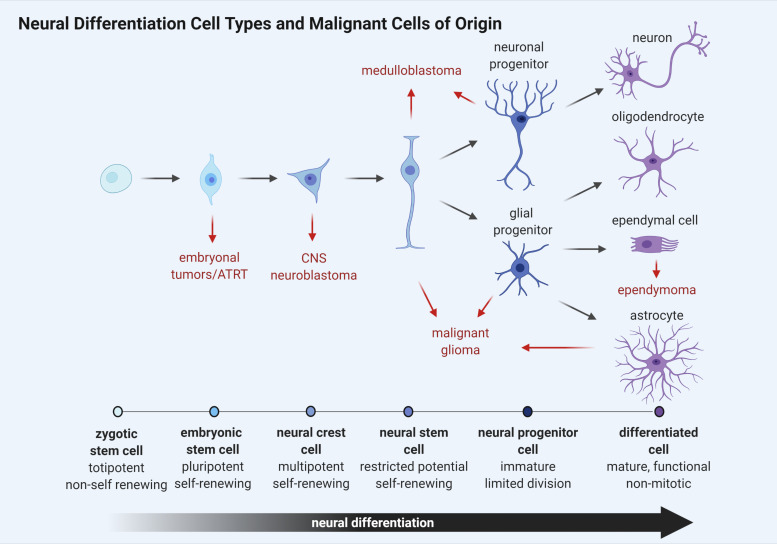
Schematic representation of neural cell differentiation in development. As cellular differentiation ensues, proliferative capacity is inversely reduced until terminal differentiation is reached and cells become senescent. Brain tumor subtypes are listed adjacent to the cell type most closely resembling a putative cancer cell of origin (red arrows).

While the identification of a cell of origin for brain tumor subtypes has yet to be entirely defined, many researchers agree that maintenance and continued dissemination of brain tumor cells depends upon a self-renewing, stem cell-like population of neural progenitors. Stem cells represent a heterogeneous population of undifferentiated cells with the potential to develop into numerous cell types (Figure 2). These cells are primarily distinguished from other cells in their capacity to self-renew through cell division without a loss of proliferative capacity with each successive division. Furthermore, stem cells can differentiate into tissue or organ-specific cells under physiologic or experimental conditions, giving them properties with strong biological resemblance to the embryonic stem cells found in the inner cell mass of early *in utero* development. Remarkably, the capacity for self-renewal through cell division can give rise to cancer phenotypes when altered or aberrantly controlled.

Like normal stem cells, cancer stem cell (CSC) division is asymmetric, giving rise to two populations. One daughter cell retains the property of self-renewal like the parent, while the other daughter cell retains the ability to differentiate but not self-renew, giving rise to tumor cell populations (Soltysova et al., 2005). These CSCs can either arise from normal stem cells having altered proliferative pathways or from somatic cells that have acquired oncogenic mutations (Figure 2). They are often resistant to drug therapy and are often causally related to oncologic relapse or recurrence following chemotherapy-induced remission. CSCs uniquely possess the ability to seed at distant or remote physiological sites, giving rise to metastatic disease and are regulated by many of the same developmental pathways that control normal stem cell development including SHH, WNT, and notch receptor (NOTCH) (Yu et al., 2012).

Cancer stem cells of CNS origin have the ability to grow on non-adherent surfaces and give rise to spherical cell colonies known as neurospheres. CD133-positive cells isolated from neurospheres are capable of self-replication, giving rise to new neurospheres, which are then able to differentiate into principal cell types of the CNS including neurons, astrocytes, and oligodendrocytes (Jordan et al., 2006). The CD133 marker is also expressed by normal stem cells of the CNS and is specifically correlated with the properties of self-renewal, proliferation, and differentiation (Uchida et al., 2000).

Cancer stem cells have also been isolated from gliomas in which they are thought to sit at the top of a cellular hierarchy within tumors (Singh et al., 2004), reviewed in Prager et al. (2020). These CSCs, known specifically as glioma stem cells or GSCs, have similar properties to neural stem cells (NSCs) and express several markers associated with stemness such as SOX2, nestin, homeobox protein NANOG, and oligodendrocyte transcription factor 2 (OLIG2; Bruggeman et al., 2007; Stiles and Rowitch, 2008; Po et al., 2010; Zbinden et al., 2010). Moreover, they can be induced to differentiate into both neuronal and glial lineages, thus contributing to cellular diversity within the tumor (Singh et al., 2004; Lee et al., 2006). The presence of GSCs gives credence to the notion that while differentiated cells can populate the majority of the tumor mass, a second group of slowly dividing stem cells lies at the center of the tumor’s capacity to self-renew and propagate to secondary sites. Owing to their robust stem-like qualities, CSCs, including GSCs, are thought to be able to rapidly respond to targeted therapeutic approaches and thus represent a resistant and therapeutically challenging group of cells that support both the processes of tumor growth and tumor resistance and recurrence (Singh et al., 2003; Bao et al., 2006; Bleau et al., 2009).

Efforts to identify a definitive marker for GSCs have proven challenging. Initially, GSCs were isolated from CD133-positive cells and demonstrated a potent ability to reconstitute tumors in xenograft models, although CD133-negative cells have also been reported to possess stem-like properties that mechanistically support tumor growth (Beier et al., 2007; Joo et al., 2008). Additional studies have further elucidated GSC signatures, noting enrichment in a number of cellular markers including CD15 (stage-specific embryonic antigen-1, SSEA1), CD44, CD24, integrin α6, and sex determining region (SRY) box2 (SOX2) (Ligon et al., 2007; Son et al., 2009; Lathia et al., 2010; Annovazzi et al., 2011; Jin et al., 2013; Neftel et al., 2019). Interestingly, recent scRNAseq data has emerged and has challenged the notion that a single GSC population may give rise to all subsequent tumor cells. Instead, these data suggest that GSC populations have a degree of heterogeneity wherein subsets of GSCs can be found in a variety of discrete cellular states, each of which is associated with distinct cellular differentiation and tumorigenic potentials (Patel et al., 2014; Neftel et al., 2019; Suva and Tirosh, 2020).

Cells with stem-like properties have also been isolated from a wide range of adult and pediatric brain tumors such as ependymomas and neuroectodermal tumors (Hemmati et al., 2003; Galli et al., 2004). CD15-positive CSCs identified in medulloblastomas revealed the existence of a certain population of CD15-positive cells expressing the TFs atonal bHLH transcription factor 1 (ATOH1), suggesting that the ability to propagate tumors may not only be restricted to a rigorous “stem-like” state but instead may result from cycling fate-restricted progenitors that give rise to tumor initiation and growth (Schuller et al., 2008; Yang et al., 2008; Ward et al., 2009; Vanner et al., 2014). To this end, Zhang L. et al. (2019) showed that OLIG2-positive progenitor cells are sufficient to drive formation of the SHH-activated subtype of medulloblastoma, noting that these cells show expression characteristics reminiscent of oligodendrocyte precursor cells (OPCs). These results are consistent with previous reports that link OLIG2-positive OPC-like cells to cells of origin in other CNS tumors, including GBMs (Ligon et al., 2007; Lu et al., 2016). Intriguingly, other fate-restricted cell populations have been linked to tumor initiation and have been shown for astrocytes in astrocytoma and chondroitin sulfate proteoglycan 4 (NG2)-positive OPCs in oligodendrogliomas (Chow et al., 2011; Zong et al., 2015).

Collectively, the robust body of literature examining the role of stem cells and CSCs in CNS tumors has demonstrated an increased appreciation for the developmental paradigms in cancer biology. The reliance of tumors on stem cell renewal and differentiation potential strongly parallels the cellular growth and differentiation pathways found in the developing CNS. In particular, the identification of CSCs endowed with properties of self-renewal and the ability to propagate tumor progression has emphasized the need for further investigation and understanding of the molecular mechanisms that drive CNS stem and progenitor cell proliferation and differentiation. Shifting emphasis away from defining common mechanisms amongst tumor categories and refocusing on defining parallels between developmental paradigms and tumorigenesis remains a promising avenue of pursuit for developing targeted therapeutic strategies for particular CNS cancer types brain tumor subtypes.

## Early Embryogenic Pathways and Morphogens

The CNS is derived from the neural plate which folds to form a neural tube in a delicately timed embryonic process known as primary neurulation. This tubular structure will become the basis for the entirety of the brain and spinal cord. Following neurulation, neural crest cells that will give rise to the peripheral nervous system are disconnected from the neural tube, which serves as a primitive embryonic precursor of the CNS. The processes of neural induction, migration and differentiation within the developing nervous system are initiated and maintained by the presence of cell signaling molecules and growth factors secreted from specific embryonic loci. A select family of signaling molecules, known as morphogens, diffuse throughout tissue across varying concentration gradients to help direct the cell fate and differentiation processes of tissue patterning. SHH, bone morphogenetic proteins (BMPs), WNT ligands, NOTCH ligands, chordin (CHRD), Hippo signaling members, and retinoic acid (RA) are each morphogen signaling families that significantly contribute to these neurogenic and gliogenic events. These organizational signals first function to pattern the developing neural tube. As neurodevelopment continues, a series of concatenated or stochastic genetic and epigenetic signatures produce iterations of cellular differentiation and proliferation that culminates in highly diverse populations of CNS cells with a variety of mature CNS functions. The expression patterns generated during the first stages of embryogenesis establish expression domains that lead to the establishment of progenitor regions. These regions are further refined spatially and are maintained by the expression of specific sets of TFs. The combinatorial expression and interaction of the transcriptional factors within the progenitor domains specify cell fate and help to establish cell identity (Briscoe and Novitch, 2008). Both spatial and temporal mechanisms must operate without miscalculation to generate distinct neuronal and glial subtypes; errors in these processes have been linked to a number of congenital CNS abnormalities, the most common of which is spina bifida resulting from a failure of the neural tube to close completely (Nikolopoulou et al., 2017).

An abundance of research over the past half century has demonstrated recurrent and essential roles for many of these early embryonic signaling pathways in the context of CNS tumors. In the following sections, we will summarize the literature pertaining to the most well-studied of these pathways and will highlight seminal work in the fields of developmental and cancer biology that has helped shape the developmental lens through which we can view brain tumors.

### Sonic Hedgehog Signaling Pathway

Sonic hedgehog (SHH) is a morphogen secreted from the notochord in the developing embryo and is responsible for patterning of the limbs (Currie and Ingham, 1996), midline structure of the brain and spinal cord (Lewis and Eisen, 2001), and teeth (Dassule et al., 2000). In particular, SHH signaling is essential for proper development of the cerebellum and regulates expansion of granule cell precursors (GCPs) (Corrales et al., 2004). The SHH signaling pathway involves a number of downstream signaling molecules including protein patched homolog 1 (PTCH1), smoothened frizzled class receptor (SMO), the glioma-associated oncogene family zinc finger proteins (GLIs) and suppressor of fused homolog (SUFU). SHH activates the transmembrane protein SMO, which in turn signals intracellularly to activate GLI transcription factors GLI1, GLI2, and GLI3. SMO activation is regulated by inhibition of PTCH1, which normally functions to block SMO function (Goodrich and Scott, 1998; Ingham, 1998). GLI1 and GLI2 work together to positively modulate SHH signaling whereas GLI3 is thought to antagonize the SHH response (Altaba et al., 2002). SUFU functions by either impeding nuclear localization of GLI proteins or by acting as a repressor of GLI signaling (Cheng and Yue, 2008). Owing to its inhibitory regulation of GLI, SUFU is considered to have tumor suppressor functions. Aside from playing a role in tumorigenesis, mutations in SHH can cause a loss of the ventral midline in development, resulting in a failure of the forebrain to develop into two distinct hemispheres, a condition known as holoprosencephaly (Nanni et al., 1999). Additionally, mutations in SHH and PTCH1 can lead to spontaneous fetal abortion.

Longstanding evidence has implicated SHH pathway activation in the regulation of renewable adult stem cell populations (Rowitch et al., 1999), suggesting a role for SHH in carcinogenesis of the CNS. SHH signaling pathways have been reported to be active in medulloblastoma and GBM, and to a lesser extent in neuroblastoma (Shahi et al., 2008). It is thought that the SHH signaling pathway mediates GBM pathogenesis and progression via regulation of the SHH/GLI1 axis. In GSCs, specific disruption of the SHH/GLI1 axis dictates the chemoresistant and radioresistant properties of the tumor and ultimately disease prognosis (Santoni et al., 2013).

Activity of the tumor suppressor PTEN has been shown to influence phosphatidylinositol 3-kinase (PI3K) signaling, which then works in conjunction with SHH signaling to promote tumor growth and viability (Filbin et al., 2013). PTEN plays an important role in proper maintenance and expansion of neural progenitor populations, suggesting a link between mismanaged NSC proliferation and neurological cancers (Groszer et al., 2001). In human GSCs, PTEN-deficient tumors manifest a significantly higher level of PTCH1 gene expression than their PTEN-expressing counterparts (Xu et al., 2008). The expression levels of SHH and GLI1 have been noted to be significantly higher in PTEN-expressing cells than in PTEN-deficient cells and corresponds to decreased survival time in GBM patients.

In medulloblastoma, some evidence suggests that tumors may originate from the external granule layer of the cerebellum (Kadin et al., 1970). The external granule layer is a germinal zone containing SHH-regulated GCPs. Dysregulation and overactivation of SHH signaling within GCPs is therefore thought to be responsible for medulloblastoma development and progression (Marino, 2005). The presence of many SHH-related alterations in these tumors has led to the classification of a medulloblastoma subtypes including the SHH-activated group. Increased activation of SHH signaling in these tumors is thought to result primarily from mutations in PTCH1 (Pietsch et al., 1997; Raffel et al., 1997; Dong et al., 2000) or SMO, although mutations in SUFU (Taylor et al., 2002) have also been observed. Interestingly, no human cancers have been reported as a result of GLI protein mutations (Erez et al., 2002; Altaba et al., 2004). Therapeutically, successful abrogation of NPC hyperproliferation and tumorigenesis as a result of aberrant GLI expression has been achieved using the SHH signaling inhibitor, cylcopamine (Berman et al., 2002; Altaba et al., 2004). Cyclopamine acts downstream of PTCH1 and is thought to influence the balance between inactive and active forms of SMO (Taipale et al., 2000) and thus helps ameliorate a hyperproliferative state.

### BMP Signaling Pathway

Bone morphogenetic proteins (BMPs) represent a large group of cytokines with the potent ability to initiate ectopic bone formation (Hopkins, 2016). Since their initial discovery more than 50 years ago, upward of twenty BMP ligands have been identified (Chen et al., 2004b; Hopkins, 2016). As a subfamily of the transforming growth factor β (TGFβ) superfamily (Piccirillo and Vescovi, 2006), BMPs are soluble factors produced in embryonic stem cells as early as the 16-cell stage (Graham et al., 2014). It has been well established that differential BMP signaling is required for the appropriate development of primitive ectoderm and trophectoderm; similarly, expression of BMPs in extra-embryonic layers is required for proper development of the embryonic structure known as the primitive streak as well as appropriate positioning of the heart (Kishigami and Mishina, 2005). Specifically, BMP2 and BMP4 are required for initiation of gastrulation and both dorso-ventral and anterior-posterior axis formation (Kishigami and Mishina, 2005).

Bone morphogenetic proteins also regulate mesoderm and cartilage formation and help direct postnatal development of bone (Chen et al., 2004a). During neurodevelopment, BMPs contribute to the rostro-dorsal patterning of the forebrain (Rubenstein, 2011) and development of paramedial structures like the choroid plexus, dorsal midline and dorsal pallium (Fernandes et al., 2007). In NSCs, BMP is a driver of astrocytic differentiation (Hu et al., 2010; Hover et al., 2016). The interaction between BMP and its antagonist, noggin, determines the fate of OPCs and regulates acquisition of the astrocytic phenotype (Piccirillo and Vescovi, 2006).

In particular, BMP signaling is involved in regulating the transcription of genes involved in cell specification including intracellular interactions used during cellular morphogenesis, apoptosis, proliferation, and differentiation (Hover et al., 2016). During signaling, BMPs bind to a heterodimeric complex of BMP serine-threonine kinase receptors type I and II, which in turn initiate the phosphorylation of regulatory SMADs, including SMAD1, SMAD5, and SMAD8. Subsequent binding of regulatory SMADs to SMAD4 leads to translocation of the complex to the nucleus for regulation of transcription (Chen et al., 2004b; Voumvourakis et al., 2011; Hover et al., 2015). Regulatory targets, including extracellular antagonists and intracellular modulators such as SMAD6 and SMAD7, can mediate BMP activation of transcription (Hopkins, 2016; Hover et al., 2016).

A 2005 study demonstrated that BMP4 is a key regulator of tumor proliferation in GBM. Transient *ex vivo* delivery of BMP4 was shown to potently inhibit the ability of human-derived GBM cells to successfully initiate tumor formation following intracerebral transplantation, while *in vivo* delivery of BMP4 effectively blocked tumor growth and reduced associated mortality following intracerebral grafting (Piccirillo and Vescovi, 2006). Likewise, BMP7 has been shown to inhibit tumor proliferation by arresting glioma-derived cells in the G1 phase of cell cycle (Klose et al., 2011). This antiproliferative property of BMP7 has been further corroborated by *in vivo* optical imaging of luciferase-tagged glioma-derived cells that have been intracranially implanted in mice.

Bone morphogenetic protein-mediated inhibition of tumor cell proliferation has also been established in medulloblastoma cells (Grimmer and Weiss, 2008). A study conducted by Zhao et al. demonstrated that reduced proliferation using BMP2 and BMP4 treatment of cerebellar GCPs is a result of proteasome-mediated degradation of ATOH1, a highly expressed TF in cerebellar GCPs. Inhibition of self-renewal and induction of differentiation are also responsible for reduced proliferative capacity within CSC populations (Caja et al., 2015). Interestingly, BMP7 is the top down-regulated gene in GSCs that proves resistant to TMZ, the DNA alkylating agent used as a first line of treatment in gliomas (Tso et al., 2015). Exogenous BMP7 treatment and augmentation of BMP7 signaling in TMZ-resistant GSCs inhibits self-renewal and migratory capacity, reduces mRNA expression of CD133, MGMT, and ATP-binding cassette drug effluxing transporters and induces senescence, thus sensitizing tumor cells to TMZ treatment. BMPs have globally been implicated as inhibitors of migration and invasion in medulloblastoma cells predominantly because of the increased migratory properties following repression of BMP pathways (Merve et al., 2014).

As a 120 kDa antagonist of BMPs, CHRD is expressed as early as gastrulation to regulate dorso-ventral patterning (Larrain et al., 2000; Anderson et al., 2002). It consists of four cysteine-rich domains which have a dorsalizing role in *Xenopus* embryo assays and bind the antagonist, BMP (Larrain et al., 2000). During embryogenesis, CHRD is expressed in the prechordal plate and anterior neural ridge (the organizing centers for rostral development) and promotes the development of the mammalian head (Anderson et al., 2002). Regulation of BMP signaling through CHRD is required for the formation of the primitive streak and stabilization of expressed neural markers on induced neural cells (Streit et al., 1998). Due to its role in dorsalization during gastrulation, defects in CHRD expression lead to congenital head and neck malformations including Velo-Cardio-Facial and DiGeorge syndromes (Bachiller et al., 2003). CHRD also regulates the bioavailability of BMP for inducing the differentiation of mesenchymal cells into bone and cartilage cells (Reddi, 2001). In addition to CHRD expression in prenatal development, Mikawa and Sato have also shown its expression in most neurons and neuropil of the cerebellum and superior colliculus in adult brain (Mikawa and Sato, 2014). CHRD plays a role in hippocampal plasticity and spatial learning in adult brain by enhancing the presynaptic neurotransmitter release from hippocampal neurons, resulting in enhanced long-term potentiation (Sun et al., 2007). Expression of CHRD has also been shown in trigeminal nuclei, particularly in dendrites, establishing its role in its regulation of dendritic morphology and synaptic homeostasis in adult trigeminal system (Hayashi et al., 2016).

From a disease standpoint, CHRD seems to impart a neuroprotective effect in injured or diseased adult brain (Jablonska et al., 2010), which is likely due to CHRD-mediated contributions to cellular differentiation that can facilitate repair in the CNS. To this end, CHRD is responsible for maintaining the lineage plasticity of progenitor cells in the subventricular zone as shown by the shift in glutamic acid decarboxylase 65 (GAD65)-positive and doublecortin (DCX)-positive progenitors from neuronal to glial fates during demyelination (Jablonska et al., 2010). Since CHRD antagonizes BMPs, it serves as a potential therapeutic target to promote the differentiation of GSCs, thus inhibiting the progression of gliomas (Videla Richardson et al., 2016).

### Transforming Growth Factor β Signaling Pathway

Like BMPs, Transforming Growth Factor β TGFβ signaling members belongs to the TGFβ superfamily that includes a number of growth differentiation factors with multiple roles in neurodevelopment and tumorigenesis. These pathways have been shown to regulate cell proliferation signaling cascades and serve as integral modulators of cellular differentiation, morphogenesis, extracellular matrix (ECM) formation, and other key functions in a wide variety of cells (Golestaneh and Mishra, 2005). Induction of TGFβ signaling can either occur through small mothers against decapentaplegic (SMAD)-dependent (canonical) or SMAD-independent (non-canonical) signaling cascades, two divergent pathways culminating in contrasting biological activity. Activated TGFβ can bind to three receptor classes of serine-threonine kinase receptors – TGFβ receptor I, II, and III (TGFRI, TGFRII, and TGFRIII) – which enhances TGFβ2 binding to TGFRβ3. Activation of TGFRI requires TGFβ binding to TGFRII, forming a tetrameric complex with the dimeric TGFβ ligand (TGFβ1, TGFβ2, or TGFβ3) and the two receptors (Derynck and Zhang, 2003). TGFRI is then activated by TGFRII via the phosphorylation of its glycerine-serine domain, and as a result, is able to phosphorylate intracellular SMAD3. Translocation of activated SMAD complex into the nucleus leads to downstream transcriptional changes and modulated gene expression (Derynck and Zhang, 2003). TGFβ has also been shown to activate a number of SMAD-independent signaling cascades including the Ras/MAPK/Erk, JNK, Rho/ROCK, PI3K-Akt, and PP2-S6 kinase pathways (Derynck and Zhang, 2003).

Dysregulation of TGFβ signaling or its downstream pathways have been associated with the pathogenesis of multiple CNS tumors, including adult gliomas such as GBM (Kaminska et al., 2013), pediatric high-grade gliomas like diffuse intrinsic pontine glioma (DIPG; Caja et al., 2015), and medulloblastomas (Kool et al., 2008). Recent reviews have detailed the role of TGFβ signaling in proliferation, invasion, angiogenesis, immune responses, and therapeutic treatments for malignant gliomas (Han et al., 2015; Birch et al., 2020; Kaminska and Cyranowski, 2020). Most notably, TGFβ has been implicated in glioma progression as a regulator of cellular proliferation, infiltrative growth, angiogenesis, and immune suppression (Fogarty et al., 2005). Although TGFβ functions as a tumor suppressor by restricting glial cell growth during development, this inhibitory capacity is lost during gliomagenesis (Fogarty et al., 2005). As tumorigenesis continues, glioma cells begin the production and secretion of TGFβ, which further exacerbates dysregulation of cell proliferation and invasion pathways, suppression of antitumor immunomodulatory responses, and importantly, the developmentally-linked oncogenic hallmark of epithelial-to-mesenchymal transition (EMT; Huber et al., 2005). This cascade of events is exacerbated by increased expression levels of TGFβRs by GSCs, which have been shown to depend upon TGFβ signaling for self-renewal and maintenance of a dedifferentiated cell state (Ikushima et al., 2009; Penuelas et al., 2009). TGFβ activity also modulates the expression of matrix metalloproteinases (MMPs) that digest and degrade the surrounding ECM, thereby facilitating invasion of malignant cells into adjacent neuropil (Rooprai et al., 2000). Moreover, TGFβ1 induces the expression of integrins that directly promote the capacity of glioma cells to migrate (Platten et al., 2000).

In the search for better therapeutic treatments for malignant glioma, an important consideration is the unintended activation of wound responses and subsequent induction of TGFβ signaling following chemotherapy, radiation or surgical resection of tumors. To this end, activation of TGFβ signaling has been observed in patient-derived GBM cell lines that have been treated with TMZ or radiation and results in enhanced migration and infiltration (Barcellos-Hoff, 1993; Canazza et al., 2011; Desmarais et al., 2012; Zeng et al., 2017). Owing to its robust involvement in the maintenance of embryonic stem cells, NSCs, and GSCs and their self-renewal and multipotency capacities, TGFβ signaling may play a direct role in chemotherapeutic and radiotherapeutic resistance and recurrence in gliomas (Watabe and Miyazono, 2009).

### WNT Signaling Pathway

Within the developing CNS, wingless-type MMTV integration site family (WNT) proteins work to regulate cell proliferation and its expression is active both during early phases of development as well as during later stages of organ and tissue growth. WNT signaling can occur through the WNT/β-catenin-dependent canonical pathway or through the WNT/β-catenin independent pathway. In the WNT/β-catenin canonical pathway WNT binding to its target receptors leads to intracellular accumulation and nuclear translocation of β-catenin, a coactivator of TFs encoded by the CTNNB1 gene that regulates expression of pro-survival and cell proliferation genes (Bengoa-Vergniory and Kypta, 2015). Non-canonical WNT signaling also controls a number of downstream TFs and cytoskeletal and cell adhesion regulators and functions through a group of proteins known as secreted frizzled-related proteins (SFRPs) (Komiya and Habas, 2008). During development, WNT signaling is essential for the proliferation and self-renewal capacity of cells and plays a large role in cell fate determination as well as primary axis formation and organogenesis (Komiya and Habas, 2008). During early development of the CNS, a WNT-mediated protein gradient is established in the developing embryo that results in increased signaling activity in the posterior region and decreased signaling in the anterior region. This polarity generated by a disparity in downstream signaling cascades leads to the proper designation of the anterior-posterior axes of the neural plate (Kiecker and Niehrs, 2001). This gradient of WNT signaling produced along the anteroposterior axis is particularly important for the formation of the anterior head structures as well as for neuroectodermal patterning (Yamaguchi, 2001). Consequently, mutations in the WNT proteins and subsequent changes to WNT-mediated signaling pathways can result in improper anterior-posterior patterning of the early CNS (Mulligan and Cheyette, 2012). In addition to its role in global patterning of the developing CNS, the signaling of WNT proteins has been shown to both inhibit and promote neurite outgrowth to regulate dendritic and axonal genes, respectively, during neurodevelopment (Selvaraj et al., 2015).

Wingless-type proteins are amongst some of the most well-studied in the context of brain tumors, and have been shown to bidirectionally support tumor progression and oncogenesis in a context-dependent manner. WNT signaling in the context of brain tumors has been most extensively studied in medulloblastoma, GBM and other astrocytoma subtypes (Manoranjan et al., 2012). In medulloblastoma, the WNT-activated subtype results from mutations to the WNT/β-catenin signaling pathway and is commonly associated with CTNNB1 or germline adenomatosis polyposis coli (APC) mutations (Helgager et al., 2020). Unlike the SHH-activated subtype of medulloblastoma, the WNT-activated subtype confers the best prognosis, although it is also the least commonly occurring variant. WNT interactions with the secreted frizzled-related proteins (SFRPs) function as signaling modulators of cell growth, regulation, and differentiation pathways. Their effects are thought to be exerted through both direct molecular interactions with WNT proteins, as well as antagonizing effects generated by interactions with other SFRP proteins. Abnormal activation of the WNT pathway has been linked to tumor formation both via the activation of effector molecules and the loss of tumor suppressor function (Shi et al., 2007). Although SFRP genes are known to be tumor suppressors in the WNT pathway, these genes can be silenced by methylation. Loss of WNT pathway inhibition due to SFRP gene silencing has been shown to be an additional mechanism that can lead to excessive WNT signaling, and in turn overexpression of the genes in the pathway (Kongkham et al., 2010). In particular, SFRP1 is in a class of SFRP genes that affect cell growth. Its role as a tumor suppressor is often lost in patients with cancer (Nicot, 2015).

In glioma, experimentation with SFRP1 as an inhibitor of the WNT pathway led to the discovery of its role as a direct target of miR-32, which is highly upregulated in invasive glioma cells (Delic et al., 2014). In GBM, recent evidence has also emerged showing that WNT proteins have a potent regulatory role in maintenance of CD133-positive CSCs (Shevchenko et al., 2019) and thus have highlighted the potential therapeutic applications of WNT-specific therapies (Zuccarini et al., 2018). In addition to its functions in promoting GSC stemness, studies have shown that WNT signaling indirectly promotes EMT in malignant glioma through its regulation of frizzled class receptor 4 (FZD4) (Jin et al., 2011). The importance of WNT-mediated EMT activation in disease outcome has been clearly illustrated by use of a WNT/β-catenin inhibitor, XAV939, which successfully suppresses EMT-driven glioma cell invasiveness (Lee et al., 2016). Moreover, WNT5A activation via the non-canonical branch of WNT signaling has been shown to directly regulate GBM cell migration through stimulation of matrix metalloprotease 2 (MMP2). Suppression of WNT5A signaling causes a reduction in MMP2 expression thereby suppressing cell invasion and migration in human glioma cell lines (Kamino et al., 2011).

### Notch Signaling Pathway

Like its SHH and WNT counterparts, notch receptor (NOTCH) signaling is an essential contributor to early neurodevelopment with an important role in the maintenance of NSC populations (Hitoshi et al., 2002; Lasky and Wu, 2005). NOTCH signaling via binding of its ligands like delta-like (DLL1) and jagged (JAG) drives cell fate determination in both developing cells of the CNS and CSCs (Koch and Radtke, 2007). Early NOTCH signaling assists in neural versus epidermal lineage commitment (Cau and Blader, 2009) and most potently drives cell fate decisions in NSCs (Yoon and Gaiano, 2005). Studies have shown that NOTCH maintains its potency in the adult brain by modulating NSCs and additionally functions postnatally to direct migration, morphology, and synaptic plasticity (Ables et al., 2011).

NOTCH signaling in cancer mirrors its functions in neurodevelopment, impacting cell fate and maintenance of CSCs (Stockhausen et al., 2010). Activators of the NOTCH signaling pathway are also known to be involved in the cellular response processes to hypoxia and neoangiogenesis, which are commonly encountered in human gliomas and thought to contribute to the pathophysiology of disease progression (Fischer et al., 2005). Investigations into how NOTCH pathway signaling drives self-renewal of brain CSCs and their potential to produce tumors has led to the identification of NOTCH signaling members as putative targets for the treatment of brain cancers (Zhang et al., 2008).

Preliminary experiments examining the effects of NOTCH pathway blockade in human GBM models showed a significant decrease in GBM cell proliferation both *in vitro* and *in vivo* (Chen et al., 2010). Additional *in vitro* studies using patient-derived GBM neurospheres have confirmed that NOTCH signaling imparts tumorigenicity in GBM cells by showing the properties of cell growth and stem-cell like features is in part attributed to activation of NOTCH pathway proteins (Kristoffersen et al., 2013).

Research has also shown that interaction between epidermal growth factor receptor (EGFR) and NOTCH pathway proteins may function to promote proliferation of cancer cells. EGFR is involved in a number of cell processes including proliferation, migration, and cell survival (Stockhausen et al., 2010). Studies investigating a NOTCH-EGFR interplay have identified EGFR as an important downstream molecular target of NOTCH signaling and have reported EGFR overexpression in 40–50% of GBM cases (Zhang et al., 2008). Not only is there a strong correlation between significant overexpression of both NOTCH proteins (NOTCH1) and EGFR in GBM tissue but moreover, a functional association between expression of these genes and patient survival has also been identified (Xing et al., 2015). The importance of NOTCH-EGFR interactions in GBM has been further bolstered by reports showing that NOTCH1-driven upregulation of EGFR also interacts with p53 in glioma cells (Purow et al., 2008).

### Retinoic Acid Signaling Pathway

Perhaps the most notorious of the oncogenic neurodevelopmental factors are retinoids (vitamin A), including the metabolite all-*trans*-RA, more commonly referred to as RA. As an essential component of early embryonic development, RA is widely recognized for its teratogenicity most frequently encountered with sustained use of retinoid pharmaceuticals during pregnancy (Ross et al., 2000). RA is a potent morphogen in embryogenesis but also holds important postnatal homeostatic functions. In order for RA to exert its effects, retinol must first be converted to RA and enter the cell via binding to retinol-binding protein 4 (RBP4). RBP4 interacts with its membrane receptor STRA6, allowing retinol to enter into the cytoplasm (Maden, 2007). In embryos, retinol is metabolized to retinaldehyde, and finally to RA (Maden, 2007). RA can act via autocrine or paracrine signaling, though the mechanism of the latter is poorly understood. In autocrine signaling, RA is transported to the nucleus with help from cellular RA-binding protein 2 (CRABP2), where it then binds to a transcription complex consisting of a RA receptor-retinoic X receptor (RAR-RXR) heterodimer. These TFs then bind to a DNA sequence called a RA-response element (RARE), leading to the transcription of over 500 genes, many of which are crucial for neuronal differentiation during the development of the CNS (Maden, 2007). Additionally, RA, along with WNTs, fibroblast growth factors (FGFs), SHH, and BMPs, contributes to the anteroposterior and dorsoventral patterning of the neural plate and neural tube, particularly in the development of the posterior hindbrain and anterior spinal cord (Maden, 2007). Retinoids also modulate genomic and postgenomic expression, exert antiangiogenic effects, and interact with protein kinase C pathways.

Retinoids have long been used in its many forms as chemotherapeutic agents in hematologic malignancies, with more recent studies documenting some benefit for GBM and medulloblastoma (Hallahan et al., 2003; Haque et al., 2007). Impaired RA signaling has been found to reduce cell differentiation and promote uncontrolled proliferation, the consequences of which have been observed in GBM (Campos et al., 2015). Specifically, 13-*cis*-RA has been used for the treatment of recurrent GBM although significant systemic toxicity has been noted with its use (See et al., 2004). In human medulloblastoma cells, use of RA has been shown to induce cell growth arrest as evidenced by inhibition and decreased expression of the cell cycle markers cyclin D 1 (CYCD1) and MYC proto-oncogene bHLH transcription factor (CMYC) (Chang et al., 2007). Notably, the phase III treatment study of pediatric neuroblastoma using 13-*cis*-RA differentiating therapy showed that high-dose pulse therapy given following completion of intensive chemoradiotherapy significantly improved event-free survival in high-risk subtypes of the disease (Reynolds et al., 2003). While the efficacy of retinoid therapy for neuroblastoma is clear, approximately 50% of patients fail to respond to treatment or become resistant during the course of therapy (Dobrotkova et al., 2019).

### SLIT/ROBO Signaling Pathway

Nearly 30 years ago, Seeger et al. (1993) identified the role of roundabout (ROBO) in providing repulsive cues for axonal extension through mutations affecting CNS axon pathway development. Following these insights, the secreted slit guidance ligands, SLITs were discovered as repellants of neuronal precursor migration from the anterior subventricular zone to the olfactory bulb (Wu et al., 1999). SLIT is produced by midline glia cells in vertebrates, is strongly expressed in the septum, and binds to the receptor ROBO expressed in the olfactory bulb (Li et al., 1999; Chedotal, 2007). Three SLIT homologs exist in vertebrates, namely SLIT1, SLIT2, and SLIT3, each consisting of four tandem leucine-rich repeat domains, laminin G-like domains, EGF-like domains, and *N*-terminus signal peptide. Four ROBO receptor subtypes have been identified in vertebrates, three of which are expressed in brain and the fourth of which is expressed by endothelial cells (Li et al., 1999; Bedell et al., 2005; Chedotal, 2007). SLIT/ROBO signaling has been conclusively shown to regulate a number of cellular processes including cellular polarity, adhesion, and cell death (Dickinson and Duncan, 2010; Ypsilanti et al., 2010) but most prominently is regarded as a key regulator of axon guidance and repulsion in the developing CNS.

An important characteristic of SLIT in the developing CNS is its prevention of axonal migration to unwanted locations (Mertsch et al., 2008; Dickinson and Duncan, 2010). Of similar importance is the ability of SLIT and ROBO to regulate the targeting of axons in vertebrates and invertebrates (Chedotal, 2007). SLIT2 is involved in stimulating the axon collateral branches formation in the dorsal root ganglion and also has roles in the arborization of central trigeminal sensory axons in rodent brainstem (Wang et al., 1999; Ozdinler and Erzurumlu, 2002). The binding of SLIT to ROBO leads to actin reorganization mediating cell motility and is enhanced by the presence of heparin sulfate proteoglycans (Ronca et al., 2001). This interaction defines cell dissociation, migration coordination and anchorage through collective movements of cells (Friedl and Mayor, 2017). Additionally, the SLIT/ROBO pathway promotes interactions between E-cadherin and β-catenin at the plasma membrane, thereby promoting cell adhesion (Dickinson and Duncan, 2010). In fly, ROBO2 and ROBO3 regulate the differentiation of serotonergic neurons, while in vertebrates, ROBO1 plays an important role in neuronal differentiation (Connor and Key, 2002; Couch et al., 2004).

Cellular functions regulated by SLIT/ROBO during development are often dysregulated in neoplastic transformation (Dickinson and Duncan, 2010). SLIT and ROBO have been shown to regulate neoangiogenesis during tumor formation (Liao et al., 2010) and studies have shown that SLIT/ROBO play dual roles in cancers, serving both as tumor suppressors and oncogenes depending on cellular circumstance (Ballard and Hinck, 2012). Specifically, SLIT2 has been shown to suppress glioma cell invasion and motility as evidenced by the reduction of SLIT2 expression in GBM (Mertsch et al., 2008). Expression of SLIT2 *in vitro* inhibits the activity of CDC42, an important Rho-GTPase family member involved in cell polarity induction during tumor cell migration (Yiin et al., 2009; Gao et al., 2013). Decreased expression of SLIT2 and ROBO1 have been shown in cancers metastasizing to the brain, including ductal carcinoma of the breast (Qin et al., 2015) and may reflect changes to EMT status. Together, SLIT and ROBO also regulate downstream hallmark pathways involved in tumorigenesis, including mTOR, VEGFR, EGFR, and HER2 both through direct and indirect signaling events (Gara et al., 2015). In both developmental and neoplastic settings, promyelocytic leukemia (PML) protein controls polycomb repressive complex 2 (PRC2)-mediated repression of SLIT proteins, which reduces cancer cell invasiveness in GBM. Additionally, these proteins form the PML/SLIT1 complex that regulates sensitivity of GBM cells to therapeutic arsenic trioxide (Amodeo et al., 2017). Overexpression of ROBO1 has also been shown to decrease GBM cell motility and overexpression of SLIT2/ROBO1 can reverse radiation-induced GBM cell migration via a mesenchymal-to-epithelial transition (MET; Nguemgo Kouam et al., 2018).

### Hippo Signaling Pathway

The Hippo signaling pathway is essential for the control of embryonic development and tissue homeostasis in multiple organ systems, including the CNS, and has recently emerged as a central element in maintaining the balance between physiologic cell proliferation and the uncontrolled cell divisions characteristic of tumorigenesis. Hippo signaling pathways have been reported as regulators of myriad physiological functions, including control of organ size, regulation of metabolism, promotion of cell differentiation, and regulation of cell proliferation (Ehmer and Sage, 2016). The key molecules upstream of Hippo include neurofibromin 2 (NF2), also known as merlin, which signals through macrophage stimulating kinases MST1 and MST2 to phosphorylate and activate large tumor suppressor kinases LATS1 and LATS2 (Liu and Wang, 2015). The activated LATS kinases interact with Mps one binder homolog 1 (MOB1) to phosphorylate yes-associated protein 1 (YAP1) and tafazzin (TAZ) effector proteins (Liu and Wang, 2015; Ehmer and Sage, 2016). YAP1/TAZ levels are essential in the control of cell proliferation; the phosphorylation of YAP1/TAZ leads to interaction with 14-3-3 protein, a mediator of nuclear removal, and subsequent cytoplasmic degradation, resulting in restricted cell proliferation and increased apoptosis (Liu and Wang, 2015; Ehmer and Sage, 2016). When YAP1/TAZ proteins are not degraded in the cytoplasm, they can interact with other neurodevelopmentally regulated proteins such as WNT, TGFβ, NOTCH, and SHH (Plouffe et al., 2015). Unphosphorylated, nuclear YAP1/TAZ promote proliferation through transcriptional regulation with the TEAD/TEF family of TFs (Ehmer and Sage, 2016). In development, kinases of the Hippo pathway are thought to function as tumor suppressors through negative regulation of YAP1 and TAZ.

Dysregulated Hippo signaling has been reported in numerous cancers, including tumors of the CNS. Overexpression of YAP1 has been observed in medulloblastomas, meningiomas, ependymomas, astrocytomas, oligodendrogliomas, and GBMs (Liu and Wang, 2015). Interestingly, loss of function mutations in the tumor suppressor protein NF2 result in increased YAP1 expression and nuclear localization, leading to development of Schwannomas and meningiomas. NF2 expression is significantly reduced in malignant gliomas (Plouffe et al., 2015) and normally functions to inhibit YAP1 by promoting LATS activation and YAP1 degradation. Mutations in NF2 therefore result in YAP1 accumulation and tumorigenesis (Liu and Wang, 2015; Ehmer and Sage, 2016). Other studies have shown that YAP1/TAZ expression is positively correlated with glioma prognosis in patients whereas LATS1/LATS2 expression correlates negatively with patient outcomes (Zhang et al., 2016). In medulloblastoma, YAP1 is significantly amplified and upregulated in SHH-activated subtypes and mediates SHH-driven NPC proliferation (Fernandez et al., 2009). More recently, studies have shown that YAP1 expression leads to upregulation of chromatin remodeler helicase, lymphoid specific (HELLS) in SHH-activated medulloblastoma and is activated downstream of SHH pathway activation through SMO, a positive transducer of SHH signaling (Robinson et al., 2019).

## Lineage-Specific Transcriptional Regulators

Transcription factors are important determinants in how tumors manifest from genetic and epigenetic alterations. The mutational burden and changes to expression levels that facilitate the progression of tumor growth through the dysregulation of proliferation and cellular differentiation are often manifest downstream of the genes in which the alterations occur, ultimately culminating in transcriptional changes that effect global cell changes. TFs are essential components of the neurodevelopmental process and function in concert with signaling factors to coordinate gene expression programs that orchestrate the precise proliferation and differentiation of vast populations of cells that make up tissues and organs. Expression of TFs during development is often a tightly controlled process under normal physiological conditions. In neoplastic conditions such as brain tumors, however, TF expression and activity can be dysregulated by various genetic and epigenetic changes including point mutations, translations, amplifications, deletions, or even extend to mutations in non-coding DNA that can affect DNA binding activity. Ultimately, this altered expression leads to deviant expression programs that engender cells with a selective cell stemness, growth or migratory advantage that is exploited by brain tumors for continued proliferation and dissemination throughout the brain.

In the following sections, we will explore what is currently known about neurodevelopmental TFs with documented roles in brain tumor initiation, malignant transformation and progression. We will pay particular attention to those TFs implicated in cell fate and differentiation in the developing brain and how dysregulation of these TF programs following acquisition of a mutational load in brain tumors may serve to drive differential outcomes by driving TF-specific genetic programs and global expression changes.

### PAX Genes

The paired box (PAX) genes constitute a family of nine developmental genes encoding nuclear TFs with critical roles in the formation of organs and tissues (Buckingham and Relaix, 2007; Wang et al., 2008). All nine members of the PAX genes share a highly conserved PAX DNA binding domain (Wang et al., 2008) and have specific spatiotemporal expressions that are tightly regulated at discrete stages of fetal development (Muratovska et al., 2003). PAX genes uniformly function as embryogenic drivers through their regulation of cell proliferation, self-renewal, progenitor cell maintenance, resistance to apoptosis, terminal differentiation inhibition, coordination of various differentiation programs, and migration of embryonic precursor cells (Moscoso and Sanes, 1995; Lang et al., 2007; Wang et al., 2008; Blake and Ziman, 2014).

Although structurally similar, each PAX gene imparts unique downstream effects and varies in its regulatory contributions to organogenesis. PAX1, for example regulates epithelial differentiation within and development of the thymus (Mansouri et al., 1999), whereas PAX2 has been shown to regulate the response of kidney mesenchyme to induction (Dressler, 1995), and is involved in embryogenesis of the hindbrain (Eccles, 1998) and epithelial differentiation within the urogenital tract (Burger et al., 2012). PAX3 has strong and preferential expression in neurodevelopment with the synthesis of PAX3 occurring in the dorsal neural tube (Chi and Epstein, 2002), and regulatory effects playing a critical role in both fate determination of neural crest cells and their differentiation into enteric and peripheral ganglia, Schwann cells and melanocytes (Nelms and Labosky, 2010). Coexpression of PAX3 with PAX7 directs differentiation of myogenic progenitor cells into skeletal muscle fibers (Buckingham and Relaix, 2007), whereas PAX6 is required for development of the eyes and nose by directing the formation of lens placodes from surface ectoderm and ectoderm placodes for nasal cavities (Grindley et al., 1995). Similarly, PAX8 directs the development of follicular cells in the thyroid gland from thyroid diverticulum and also regulates the transcription of thyroperoxidase and thyroglobulin (Chi and Epstein, 2002).

Within the field of glioma biology, PAX3 has been established as a regulator of GSCs through its modulation of glial fibrillary acidic protein (GFAP) expression (Su et al., 2016). PAX3 inhibits expression of GFAP by binding its promoter site, and accordingly, overexpression of this TF promotes the differentiation of GSCs (Su et al., 2016). PAX8 has also been shown to promote gliomagenesis through expression of telomerase catalytic unit and RNA component genes in human glioma cell lines. The link between PAX8 and telomere maintenance highlights a potential therapeutic application for inhibiting proliferation of tumor cells through telomere shortening (Chen et al., 2008). Likewise, functional data showing overexpression of PAX2 in hindbrain and cerebellar development as a driver of medulloblastoma has implicated PAX2 as a proto-oncogene (Burger et al., 2012). Interestingly, PAX5, along with its paralogues PAX2 and PAX8, has been shown to downregulate the expression of tumor suppressor gene, p53 through binding to its untranslated first exon and thus has proto-oncogenic functions in tumorigenesis (Stuart et al., 1995). Dysregulated expression of PAX5 in undifferentiated medulloblastoma cells has further established PAX5 in tumor cell proliferation (Kozmik et al., 1995).

### SOX Genes

The sex determining region Y (SRY) box family of transcription factors (SOXs) is composed of 20 members containing a related high motility group (HMG) DNA binding domain. They are further categorized into 8 subgroups based on sequence similarity of the HMG group, which tend to share biochemical properties and often demonstrate overlapping expression patterns and functional redundancy. The SOX group in its entirety serve as developmental regulators with functions in many organs including CNS tissues. In particular, they are important for stem cell maintenance and have been attributed roles in tissue regeneration and tumorigenesis (Kamachi and Kondoh, 2013). Importantly, differential expression of SOX genes (SOX4, SOX9, and SOX11) in pediatric brain tumors has been used as prognostic marker for disease progression and outcomes in ependymoma and medulloblastoma (de Bont et al., 2008).

SOXB1 transcription factors are expressed early in embryonic development and are involved in NSC maintenance; overexpression of these TFs is thus not surprising in the context of brain tumors. Expression of SOX1 in human glioma is heterogeneous with high expression of SOX1 conferring poor prognosis (Garcia et al., 2017). SOX1 expression was enriched in patient-derived GSCs and its expression diminished in GSCs induced to differentiate, suggesting that SOX1 may be important for maintaining the undifferentiated state. Inhibition of SOX1 in glioma cells leads to decreased proliferation, self-renewal, and reduced capacity to grow *in vivo* (Garcia et al., 2017). Overexpression of SOX1 in GSCs only moderately increased cell growth and proliferation. Further, overexpression in differentiated glioma cells weakly induced neurosphere formation and stem cell marker expression and failed to induce tumor growth in a xenograft model (Suva and Tirosh, 2020). Therefore, it appears that SOX1 is essential for maintaining stem cell renewal, but not sufficient to promote tumor initiation.

SOX2 is an important regulator of early embryogenesis and contributes to the pluripotency of certain stem cell subsets (Avilion et al., 2003). In the adult nervous system, SOX2 is expressed in NSCs and undifferentiated precursor cells (Wegner and Stolt, 2005), and in cancer SOX2 serves as a marker of proliferating or undifferentiated cells in human malignant gliomas, including pediatric gliomas, and ependymomas (Ferletta et al., 2007; Phi et al., 2008; Annovazzi et al., 2011). SOX2 is also overexpressed or amplified in oligodendrogliomas and GBMs, and interestingly appears to be more highly expressed in the SHH-activated subgroup of medulloblastomas, perhaps suggesting a link between SHH and SOX2 signaling in a neoplastic context (Phi et al., 2010; Annovazzi et al., 2011; Ahlfeld et al., 2013). With respect to survival, SOX2 expression is positively correlated with brain tumor malignancy grade and confers poor clinical outcomes (Sutter et al., 2010; Mansouri et al., 2016). Mechanistically, SOX2 is necessary for maintaining GSC properties in GBM and medulloblastoma cells, but is not sufficient to support self-renewal properties (Alonso et al., 2011; Berezovsky et al., 2014) again highlighting a role for TFs in tumor progression but not tumor initiation. While SOX2 is highly expressed in the proliferating populations of some brain tumors it has also been shown that SOX2-positive cells in brain tumors coexpress GFAP, indicating that SOX2 may serve as a selective marker of tumor cells deriving from or assigning to a glial lineage rather than a marker of all neoplastic cells (Phi et al., 2008).

The role of SOX3 has been studied to a lesser extent in brain tumors although it is notably expressed in developing NSCs and in a subset of mature hypothalamic neurons (Bylund et al., 2003). SOX3 expression was found to be increased in a subset of primary GBM samples and in patient-derived GSC and its overexpression in glioma cells results in increased proliferation, migration, and invasion (Marjanovic Vicentic et al., 2019).

The SOXC TFs include SOX4 and SOX11, which are initially coexpressed in differentiating NPCs during early embryonic development with their expression patterns become more spatially divergent within the CNS as development ensues (Cheung et al., 2000). Studies using SOX11 deficient NPCs have demonstrated that SOX11 is necessary for both embryonic and adult neurogenesis (Wang et al., 2013), suggesting that its expression facilitates loss of stemness and acquisition of a more differentiated neuronal phenotype (Hide et al., 2009). To this end, studies have shown that SOX11 is overexpressed in gliomas and medulloblastoma and that high expression in the former is associated with positive outcomes (Lee et al., 2002; Korkolopoulou et al., 2013; Czapiewski et al., 2016). To the contrary, loss of SOX11 expression correlates with a significant decrease in survival, perhaps owing to loss of differentiated tumor cells and retention of more CSCs.

The role of SOX4 in glioma is controversial insofar as conflicting reports exist on SOX4 activity and expression, suggesting that its function may be context dependent. While some reports suggest that increased expression of SOX4 correlates with a favorable prognostic outcome, others suggest poor overall prognoses associated with high expression (de Bont et al., 2008; Li et al., 2015). In human glioma cell lines, SOX4 inhibits growth by influencing WNT and TGF signaling pathways, as well as p53-p21 activity (Zhang et al., 2014). Interestingly, SOX4 can also interact with OCT4 to activate SOX2 expression via its enhancer region thereby maintaining the stemness properties of the GSC population, a mechanism that differs from the SOX2 self-regulating loop that dominates developmental NPC proliferation (Ikushima et al., 2011).

During development the SOXD TFs, SOX5 and SOX6, are most highly expressed in OPCs, oligodendrocytes and a subset of neurons (Stolt et al., 2006). These SOXD proteins have been shown to promote the migration of OPCs by maintaining them in an undifferentiated state thereby preventing precocious differentiation of these glial cells (Stolt et al., 2006; Baroti et al., 2016). Interestingly, SOX5 expression is lower in both glioma samples and glioma cell lines than in normal adult brain (Schlierf et al., 2007). perhaps reflecting a decrease in oligodendrocytic cell identity. Overexpression of SOX5 has been shown to inhibit proliferation in both *in vitro* experiments of human glioma cell lines and *in vivo* experiments using platelet-derived growth factor β (PDGFβ)-induced glioma in mice (Tchougounova et al., 2009). While SOX6 is expressed in gliomas and medulloblastomas, it shows differential expression levels depending on tumor subtype, with lower levels found in GBM and higher levels present in oligodendrogliomas (Schlierf et al., 2007). Owing to its predominant expression in neurodevelopment, SOX6 represents a putative tumor-specific antigen in glioma; treatment of mice with a SOX6-DNA vaccination had protective and anti-tumorigenic effects on tumor bearing mice (Ueda et al., 2004, 2008).

SOXE proteins, including SOX8, SOX9, and SOX10, are generally expressed after neural induction but before the initiation of gliogenesis. Mice lacking SOX9 have impaired specification of oligodendrocytes and astrocytes, although OPCs seem to recover at later stages of development owing to the functional redundancy of SOX9 with SOX8 and SOX10 (Stolt et al., 2003). A similar functional redundancy has been observed in SOX8-null mice as no defects in oligodendrocyte specification are observed. Mice lacking both SOX8 and SOX9, however, fail to form mature oligodendrocytes (Stolt et al., 2004, 2005). Likewise, SOX10 is critical for terminal differentiation of oligodendrocytes and myelination (Stolt et al., 2002).

Little is known about the role of SOX8 in brain tumors, although it is widely expressed in oligodendrogliomas, medulloblastomas and astrocytomas with lower expression present in GBM (Cheng et al., 2001; Tchougounova et al., 2009; Azar et al., 2018).

SOX9 is strongly expressed in malignant gliomas and its upregulation is associated with higher tumor grade and worse survival outcomes (Wang et al., 2012; Gao et al., 2015; Gnerlich et al., 2019). In glioma cell lines overexpression of SOX9 stimulates migration, invasion, and the EMT process via the activation of the WNT/β-catenin signaling pathway (Liu et al., 2015). Loss of SOX9 function in these cell lines resulted in impaired cell survival and abrogated proliferation via enhanced p21^*CIP*^ expression (Wang et al., 2012; Gao et al., 2015; Aldaz et al., 2020). In a mouse model of malignant glioma, codeletion of SOX9 and POU3F2 regulatory enhancer elements in the nuclear factor IA (NFIA) locus block NFIA expression and inhibit tumorigenesis (Glasgow et al., 2017). Transcriptional regulation by SOX9 has also been associated with the SHH-activated group medulloblastomas (Swartling et al., 2012).

SOX10 is expressed in oligodendrogliomas, astrocytomas, diffuse cerebellar gliomas, and H3K27M-mutant midline gliomas (Bannykh et al., 2006) although its expression is silenced in cortical gliomas by promoter methylation. Studies in a mouse model of malignant glioma show that SOX10 is not sufficient to induce glioma tumorigenesis (Ferletta et al., 2007), likely due to the cross inhibitory relationship of SOX10 and NFIA that is present in development and is conserved during tumorigenesis in glioma (Glasgow et al., 2014).

### NFI Genes

Nuclear factor I (NFI) genes are transcription factors with CCAAT box binding domains present within their consensus sequence. NFI family members include NFIA, NFIB, NIFC, and NFIX and have distinct roles in normal development. Mutations in these genes are associated with various developmental aberrations owing to disturbances in cellular proliferation and differentiation pathways that are mediated primarily through transcriptional control of downstream NFI target genes (Gronostajski, 2000; Mason et al., 2009). NFIA and NFIB deficient mice show lethal developmental phenotypes and die at birth from lung abnormalities and profound anatomical brain defects, including corpus callosum agenesis and enlarged lateral ventricles (das Neves et al., 1999; Shu et al., 2003; Steele-Perkins et al., 2005). Both NFIA and NFIB knockout mice show pronounced defects in glial cell development (Deneen et al., 2006; Barry et al., 2008); mice lacking NFIX display enlarged lateral ventricles but do not suffer from corpus callosum anomalies (Campbell et al., 2008). Interestingly, NFIC-null mice are viable and present without developmental abnormalities (Chaudhry et al., 1997).

Roles for NFIA and NFIB as tumor suppressors in glioma have been documented, and expression of these two factors is inversely correlated with tumor grade such that higher grade tumors are associated with lower expression of NFIA or NFIB (Song et al., 2010; Stringer et al., 2016). Increased expression of NIFA or NFIB is associated with increased survival in patients with high-grade gliomas (Song et al., 2010; Stringer et al., 2016). Studies in both human and mouse glioma cell lines demonstrate that NFIA is important for glioma tumorigenesis and is mediated by regulation of p21 and p53 (Glasgow et al., 2014). Overexpression of NFIB in GBM cells induces cell differentiation and inhibits tumor growth via signal transducer and activator of transcription 3 (STAT3) signaling mechanisms (Stringer et al., 2016). In a genetic mouse model of glioma, deletion of NFIA or NFIB reduces survival of mice and increases tumorigenicity (Chen et al., 2020a,b). Moreover, ectopic expression of NFIA or NFIB in a glioma xenograft model is sufficient to promote differentiation of tumor cells.

Nuclear factor IA is lost as part of chromosome 1p31 and low expression of NFIA is associated with oligodendrogliomas (Idbaih et al., 2008; Houillier et al., 2010; Sun et al., 2014). Strikingly, overexpression of NFIA in a mouse model of oligodendroglioma can convert the tumor to an astrocytoma subtype (Glasgow et al., 2014). This function as a driver of differentiation in gliomas parallels the function of NFI factors as glial determinants during neural development and clearly illustrates how developmental paradigms are recapitulated in the context of tumor evolution.

### bHLH Genes

Proneural basic helix-loop-helix (bHLH) transcription factors are essential regulators of neural cell fate in the developing CNS and in regions of the adult NSC niches. As suggested by their namesake, bHLH factors contain a helix-loop-helix domain that is utilized for dimerization and binding to the enhancer box motif consensus sequence CANNTG. Some of the most well-studied of these TFs in development and brain tumors are OLIG2, atonal bHLH transcription factor 1 (ATOH1) and Achaete-Scute family bHLH transcription factor 1 (ASCL1). Although mutations in proneural bHLH factors are not commonplace in brain tumors, dysregulated expression levels of these TFs in some tumor subtypes may direct disease course through respective downstream changes to gene expression.

As a key TF controlling glial cell fate in the developing CNS, OLIG2 shows a diverse repertoire of functions in neurodevelopment. It is required for the generation of motor neuron populations in the spinal cord and likewise is necessary for successful generation of OPCs and their subsequent maturation in the spinal cord and cortex (Takebayashi et al., 2002; Zhou and Anderson, 2002; Zhu et al., 2012). In the absence of OLIG2, OPCs are converted to astrocytes suggesting that OLIG2 not only promotes the oligodendrocyte fate but also serves to inhibit astrocyte fate (Takebayashi et al., 2002; Zhou and Anderson, 2002; Zhu et al., 2012). OLIG2 is expressed in varying degrees in both pediatric and adult gliomas, with significant expression occurring in GSC populations of oligodendrogliomas (Lu et al., 2001; Ligon et al., 2004). A profiling study in GBM found a subset of neurodevelopmental TFs (including OLIG2 and SOX2) are sufficient to reprogram differentiated human GBM cells into GSCs, suggesting that OLIG2 has an important role in maintaining GSC stemness (Suva et al., 2014). Accordingly, deletion of OLIG2 in a mouse model of glioma results in impaired tumor growth and a shift in cellular profiles toward an astro-glial expression pattern. This shift is associated with downregulation of PDFGR and EGFR (Lu et al., 2016) and is supportive of a role for glial determinants altering the cellular constituency and fate of glioma cells.

Another important regulator of the developing brain and spinal cord cell populations is ATOH1, which is a TF expressed in progenitor populations throughout several regions of the brain and the dorsal spinal cord ([reviewed in Lai et al., 2016)]. ATOH1 is required for the proper development of dI1 dorsal interneurons in the spinal cord and the proliferation of granule cell precursors, serotonergic neurons, and respiratory nuclei of the hindbrain (Ben-Arie et al., 1996; Flora et al., 2009; Rose et al., 2009). Genetic loss of ATOH1 results in neurophysiological deficits, including a reduction in the size of the cerebellum and premature death due to dysregulated respiration resulting in apnea (Rose et al., 2009). Like many bHLH TFs, ATOH1 itself is rarely mutated in brain tumors; its expression levels, however, are reported to be dysregulated in a number of brain tumor subtypes (Fu et al., 2019). The role of ATOH1 in high-grade glioma has yet to be well-defined, although correlative relationships between high ATOH1 expression in the SHH-activated subtype of medulloblastoma have been reported (Salsano et al., 2004). The increased expression profile of ATOH1 in medulloblastoma – a cerebellar tumor subtype – is thought to reflect the prominent role of ATOH1 as a regulator of cerebellar GPCs in the developing brain (Thompson et al., 2006). While ATOH1 expression alone is not sufficient to induce medulloblastoma tumorigenesis, deletion of ATOH1 in a mouse model of medulloblastoma significantly attenuates tumorigenesis by decreasing GPC proliferation (Flora et al., 2009). Conversely, overexpression of ATOH1 in a PTCH1-deficient mouse model of SHH-activated medulloblastoma accelerates tumor progression (Grausam et al., 2017). Recently, a phosphorylated form of ATOH1 was found in human SHH-activated medulloblastoma samples and serves to stabilize ATOH1, leading to increased ATOH1-mediated activity and proliferation of tumor initiating cells (Klisch et al., 2017).

Achaete-Scute family bHLH transcription factor 1 (ASCL1) is also required for the specification of interneuron populations in the developing spinal cord and for the generation of several subsets of neuronal populations in the brain (Dixit et al., 2011; Pacary et al., 2011; Dennis et al., 2017). In addition to promoting neuronal differentiation programs, ASCL1 has been implicated in the regulation of gliogenesis as well (Nieto et al., 2001; Nakatani et al., 2013; Vue et al., 2014; Lai et al., 2016) and therefore its expression is present in both neuronal populations and a subset of glial progenitor cells (Vue et al., 2014). ASCL1 knockout mice have several neurodevelopmental defects and die shortly after birth from their deficits in neurogenesis (Nieto et al., 2001). Conditional deletion of ASCL1 following the neurogenic period has revealed that ASCL1 is important for ensuring the appropriate proportion of white matter oligodendrocytes are generated during development (Vue et al., 2014).

With respect to brain tumors, ASCL1 is expressed in GBM, astrocytoma, and oligodendrogliomas (Somasundaram et al., 2005; Rousseau et al., 2006; Rheinbay et al., 2013). *In vitro* experiments using cultured glioma cells have revealed ASCL1 is necessary for cellular proliferation via activation of WNT signaling (Rheinbay et al., 2013). In addition, glioma cell lines overexpressing ASCL1 can drive efficient conversion of these glial derivatives into neurons if induced to differentiate (Cheng et al., 2019). Notably, a subset of patient-derived GSCs express high levels of ASCL1 and retain their capacity for neuronal differentiation. Similarly, restoring ASCL1 expression to GSCs endogenously deficient in ASLC1 induces neuronal differentiation and reduces tumorigenesis by exposing the relevant chromatin regions for activation of neuronal lineage programs (Park et al., 2017). In mice, loss of ASCL1 in glioma delays tumor progression thereby increasing survival (Vue et al., 2020). In these studies, ASCL1 is shown to both directly and indirectly regulate the expression of cell cycle genes, drivers of neurodevelopment, and factors shown to regulate gliogenesis (Vue et al., 2020).

## Crosstalk Between Neurodevelopmental Signaling Pathways and Transcriptional Programs

**FIGURE 3:**
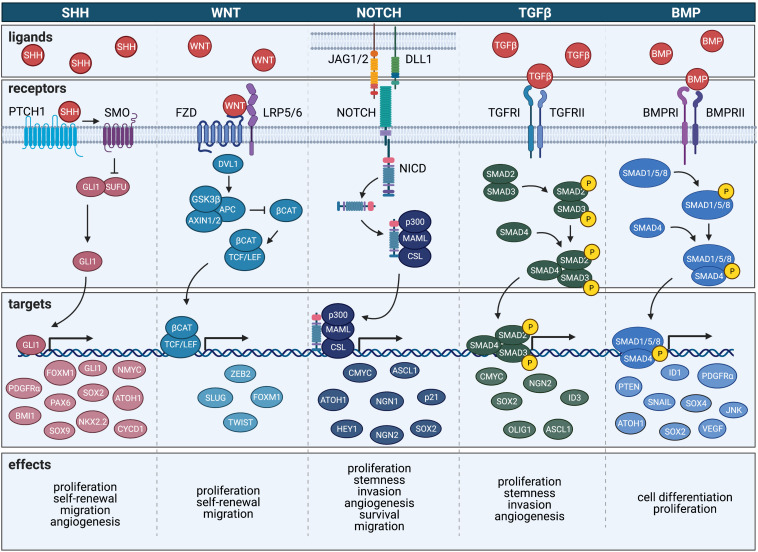
Crosstalk occurring between neurodevelopmental signaling pathways and transcriptional regulation in brain tumors.

The convergence of neurodevelopmental pathways with other signal transduction cascades in both development and disease has long been established reviewed in Brechbiel et al. (2014), Luo (2017), Pearson and Regad (2017), Neve et al. (2019), Pelullo et al. (2019) (Figure 3). In this section, we review examples of how this crosstalk between neurodevelopmental pathways (NOTCH, WNT, and TGFβ) and lineage-specific transcriptional regulators functions within brain tumors.

The most robust of these relationships is demonstrated by ATOH1-mediated transcriptional control of development and medulloblastoma progression. ATOH1 is required for development of the cerebellum where it works in a SHH-dependent manner to regulate cerebellar GCPs (Ben-Arie et al., 1997; Gazit et al., 2004). ATOH1 also regulates cerebellar GCPs development via NOTCH signaling and activation of HES family bHLH transcription factor 5 (HES5) (Gazit et al., 2004). While the induction of ATOH1 is in part self-activating, it is also regulated by BMP activity, specifically, that of BMP7 (Alder et al., 1999; Helms et al., 2000). Moreover, regulation of ATOH1 expression is mediated by a negative feedback loop that depends on HES5 (Gazit et al., 2004). During the processes of tumorigenesis, ATOH1 is required to attain GCP identity, which serves as a critical event for the formation of SHH-induced medulloblastomas (Schuller et al., 2008; Flora et al., 2009; Grausam et al., 2017). The requirement of ATOH1 for the formation of SHH-activated medulloblastoma is mediated by direct binding of ATOH1 to the SHH pathway effector, GLI2, which maintains GCPs in a SHH-responsive state (Flora et al., 2009; Ayrault et al., 2010). Collectively, these data suggest that ATOH1 acts as an oncogene in medulloblastomas; however, ATOH1-mediated tumor suppressor activity has been demonstrated in other signaling pathways and highlights the importance of context when considering these pathway interactions (Bossuyt et al., 2009a,b).

In developing glial cells, HES1 represses ASCL1 expression while ASCL1 inversely activates HES5 (Kondo and Raff, 2000). A similar pattern of cross regulation exists in malignant glioma in which ASCL1 is important for tumorigenicity and maintenance of GSCs (Zheng et al., 2010; Zhang et al., 2011; Rheinbay et al., 2013). Upregulation of ASCL1 is associated with inhibition of NOTCH signaling (Somasundaram et al., 2005). GSCs with high expression of ASCL1 are competent to differentiate in response to NOTCH inhibition, whereas low-expressing ASCL1 GSCs show reduced sensitivity to the same conditions (Park et al., 2017). In addition to its interactions with NOTCH, ASCL1 has been linked to WNT signaling using transcriptomic analyses of GSCs (Rheinbay et al., 2013). In this context, ASCL1 activates WNT signaling via induction of canonical WNT signaling target genes and sustained ectopic expression of ASCL1 leads to aberrant expression of several WNT-responsive genes.

Post-translational modifications to OLIG2 mediate migration and proliferation of both OPCs and GSCs in development and glioma, respectively (Sun et al., 2011; Hornig et al., 2013; Nevo et al., 2014; Singh et al., 2016). Whereas unphosphorylated OLIG2-positive cells are inherently invasive, phosphorylated OLIG-positive cells show increased capacity for proliferation. In tumors, the unphosphorylated form of OLIG2 drives increased patient-derived GSCs invasion via activation of the TGFβ pathway through TGFβ2 culminating in expression of EMT-associated gene (Singh et al., 2016). Expectedly, inhibition of TGFβ2 signaling suppresses unphosphorylated OLIG2-mediated invasion. This crosstalk between unphosphorylated Olig2 with TGFβ thus serves an important role dictating the proliferative or invasive properties of glioma cells.

During neurogenesis PAX6 is critical for the generation of discrete progenitor domains that occur in response to a ventral SHH signaling gradient, which leads to the production of motor neurons and ventral interneurons (Ericson et al., 1997). In both medulloblastoma and glioma cells, PAX6 expression is controlled by SHH-GLI signaling events: GLI1 activates PAX6 expression in medulloblastomas but suppresses it in gliomas (Shahi et al., 2010). In GSCs, PAX6 directly binds to WNT5A regulatory regions to induce the differentiation of GSCs into endothelial cells that in turn support the extensive vasculature that feed GBMs (Hu et al., 2016).

Similarly, the SOX family of genes can interact with SHH, TGFβ and WNT signaling pathways in both the contexts of development and tumorigenesis (reviewed in Mansouri et al., 2016). In NPCs, GLI proteins, which are downstream effectors of the SHH pathway are regulated by SOX2-dependent events. Specifically, upon induction of SHH signaling, GLI proteins cooperate with SOX2 to activate the expression of transcription factors that help specify progenitors of the ventral spinal cord, including NKX2.2 and OLIG2 (Oosterveen et al., 2012; Peterson et al., 2012). Concordantly, SOX2 expression is higher in SHH-activated medulloblastomas than it is in other medulloblastoma subtypes with SOX2 activity serving as a regulator of stemness in these tumors. In glioma, SOX2 expression promotes stemness via downstream activation of TGFβ signaling, which also promotes GSC stemness by activating SOX4 (Ikushima et al., 2009).

## Discussion

Despite the growing body of literature describing roles for developmental programs and transcriptional networks in tumor initiation and progression, methods for practically targeting these pathways have remained elusive. While differentiating therapies such as 13-*cis*-RA have been used for other cancers, including the predominantly PNS cancer of neuroblastoma, a multitude of developmental targets remain unexplored as potentially exploitable therapeutic modulators of brain tumors. In particular, TFs have been historically regarded as “untargetable” owing to the complex nature of protein-DNA and protein-protein interactions. However, recent strategies employing TF DNA binding inhibition or blockage of cofactor interactions have emerged and have begun to challenge this perspective.

More recent approaches utilizing proteasomal degradation of TFs have shown some promise (Bushweller, 2019). In addition, targeted therapies inhibiting OLIG2 in glioma are currently being tested in both laboratory and clinical settings including the use of a small inhibitory molecule that prevents the homodimerization of OLIG2 that subsequently impeding its nuclear localization (Tsigelny et al., 2017; Oasa et al., 2020). Preliminary studies in patient-derived GBM cells found that this small molecule could elicit potent anti-tumorigenic effects (Tsigelny et al., 2017) and has suggested that allosteric modulation of protein-protein interactions may be a viable approach to target transcriptional regulators in brain tumors. While TF modulation as a therapeutic modality is still in its infancy, more extensive studies elucidating details about TF protein modifications, epigenetics or 3D-chromatin landscape will help aid research and development of targeted treatments.

It is important to note that in addition to the early embryonic signaling pathways and TFs highlighted in this review, a number of developmentally-driven epigenetic aberrations have been identified in both adult and pediatric brain tumors, although these alterations seem to be more relevant to the initiation of brain tumors in the pediatric population. While the genetic mutational landscape of pediatric brain tumors appears to be sparser than adult gliomas, mutations in histone modifiers including HIST1H3B/C, HIST2H3C, and H3F3A lead to changes in methylation and acetylation profiles of histone 3. The consequences of these histone mutations result in global dysregulation, particularly overexpression of entire chromosomal regions including the expression of essential developmental regulators of cell proliferation, migration and differentiation (Schwartzentruber et al., 2012; Sturm et al., 2012; Wu et al., 2012; Bender et al., 2013). As such, clinicians and scientists have come to view these pediatric glial tumors as separate and distinct from their adult counterparts, noting that the predominant pathophysiological modality of disease progression is the inability to stop developmental programs and thus these tumors are “developmentally stuck.” In particular, the uniformly fatal pediatric glioma subtype of diffuse midline glioma (formerly DIPG) requires the presence of the H3K27M mutation for diagnosis. Notably, bivalent histone modifications featuring both H3K4me3 and H3K27me3 have been identified as an embryogenic hallmark of pluripotency in embryonic stem cells and is a feature of the PRC (Perla et al., 2020). While the H3K4me3 histone mark at gene promoters confers an active transcriptional state, the presence of the H3K27me3 histone mark is overwhelmingly repressive; thus, the duality of this bivalent histone modification inhibits expression of cell differentiation genes while keeping them poised for activation in later stages of embryogenesis. Genetic alterations, like H3K27M, occur within this complex yield tumors from cells of origin that have failed to receive proper developmental cues to mature. The phenotypes of pediatric tumors are often related to their location of origin suggesting that normal developmental transcriptional programs and signaling pathways may arise that have regional specificity which confer differential competence on tumor initiating cells in these regions. Future studies aimed at correlated how pediatric tumor location and developmental context dictates malignancy and tumorigenicity would help to determine how these events are leading to tumorigenesis.

To date, the majority of oncologic research and treatment has viewed cancer through an anthropomorphizing lens that has characterized cancer cells as malevolent and corrupt entities. Commonly used descriptors such as “aggressive” and “invasive” coupled with phrases like “hijacked” and “infiltrating” have imparted a sense of volitional evil onto these non-sentient illnesses. This context of mal-intent in which the problem of cancer has been placed has had profound effects on the ways in which we have attempted to combat these diseases. Researchers and clinicians alike have been prompted to develop anticancer treatments and therapeutics designed to “kill” and “eradicate” their targets. These hard-hitting counterattacks often employ perilous and toxic mechanisms of action, frequently leading to irreparable and fatal side effects in an attempt to contain and eliminate the disease. As a means of minimizing toxicity and damage to the patient, the fields of precision medicine and immunotherapy have attempted to trade in their weapons of mass destruction in exchange for patient-specific armamentariums of therapeutics specifically designed to target tumor cells while leaving non-diseased tissue unharmed. But these approaches maintain and perpetuate the notion of cancer as “bad” or “malicious,” and thus continue to employ tactics of destruction as a means to an end.

Recent genetic and epigenetic data have provided evidence that many cancers possess molecular fingerprints with tremendous similarities to developmental cells including stem cells and progenitors. Some oncologic diseases demonstrate such profound molecular likeness to known precursor cells that it has prompted many researchers to view cancer as a disease of development rather than a series of spontaneous and transformative events. The highly proliferative, migratory and self-renewing capacities of cells have not only become a hallmark of cancer, but additionally have been assigned as quintessential traits of the rapidly expanding cell populations characteristic of *in utero* and perinatal development. As opposed to alterations occurring in individual pathways, genes or proteins, entire developmental programs have been identified as dysregulated drivers of brain tumors. These programs include VEGF and FGF-mediated tumor derived angiogenesis (Jain et al., 2007; Acar et al., 2012), synapse formation (John Lin et al., 2017; Hatcher et al., 2020; Yu et al., 2020), regulation of organ size and growth (Liu and Wang, 2015; Ehmer and Sage, 2016), and gliogenesis (Ligon et al., 2007; Glasgow et al., 2013, 2014, 2017; Vue et al., 2020). Given these precedents, there is mounting evidence to suggest that cancer cells are perhaps undeserving of their maligned reputation as evil-doers, and instead should be viewed as persistent developmental precursors that failed to mature and are attempting to execute their molecular mandate of proliferation and migration. In this paradigm, cancer cells are not “evading” detection by the immune system, nor are they “manipulating” the microenvironment to facilitate their own expansion. They are not “recruiting” or “redirecting” resources with selfish intentions; rather, they are more simply understood as proliferating and migrating cells, commissioned to generate complex physiological structures in an environment with minimally available real estate.

If this theory of cancer as a developmentally misguided event holds true, a solution to this conundrum may be more easily reached through reeducation and redirection rather than annihilation and elimination. The task would now be to identify with which developmental progenitors these oncogenic cells most closely correlate, to determine the naturally-occurring molecular and physiological events that are responsible for regulating their developmental differentiation, and to expose cancer cells to these transformative signaling events *in vivo* to help them achieve their proper fate. These therapies aiming to direct the fate of tumor cells toward more differentiated and less malignant states– termed differentiation therapy- remains enigmatic in the case of solid neoplasms, including those occurring in the brain and CNS. For these therapies to become more feasible, continued investigations into the processes regulating cell lineage commitment and differentiation both during development and tumorigenesis must continue. While great progress in the field of brain tumor biology has been made over the last two decades, much remains to be defined before plausible treatment options for adult and pediatric brain tumors are attained and become commonplace in a clinical setting. Thus, integration of developmental biology into the current dogmas of oncogenesis, malignant transformation and tumor evolution can help to guide future research endeavors and elucidate novel therapeutic targets for these lethal malignancies of the CNS.

## Author Contributions

RC and SG researched data for the article and contributed to the writing and editing of the manuscript. Both authors have read and approved the document.

## Conflict of Interest

The authors declare that the research was conducted in the absence of any commercial or financial relationships that could be construed as a potential conflict of interest.

## Abbreviations

ACVR1, activin A receptor: type I
APC: adenomatosis polyposis coli
ASCL1: Achaete-Scute family bHLH transcription factor 1
ATOH1: atonal bHLH transcription factor 1
ATRX: alpha thalassemia/mental retardation syndrome X-linked
AXIN: axis inhibitor
β CAT: β -catenin
BMI1: B lymphoma Mo-MLV insertion region 1 homolog
BMP: bone morphogenetic protein
BMPRI, bone morphogenetic protein receptor: type I
BMPRII, bone morphogenetic protein receptor: type II
BRAF, B-Raf proto-oncogene: serine/threonine kinase
CDK4: cyclin dependent kinase 4
CDKN2A: cyclin-dependent kinase inhibitor 2 A
CHRD: chordin
CIC: capicua transcriptional repressor
CMYC: MYC proto-oncogene bHLH transcription factor
CNS: central nervous system
CRABP2: cellular retinoic acid binding protein 2
CSC: cancer stem cell
CSL: CBF-1 (RBPJ-k)/Su(H)/Lag1
CTNNB1 catenin: β -1
CYCD1: cyclin D 1
DCX: doublecortin
DLL1: delta-like canonical NOTCH ligand 1
DVL1: disheveled segment polarity protein 1
ECM: extracellular matrix
EGF: epidermal growth factor
EGFR: epidermal growth factor receptor
EMT: epithelial-to-mesenchymal transition
FGF: fibroblast growth factor
FGFR: fibroblast growth factor receptor
FOXM1: forkhead box M1
FUBP1: far upstream element binding protein 1
FZD: frizzled class receptor
GAD65: glutamic acid decarboxylase 65
GBM: glioblastoma
GCP: granule cell precursor
GFAP: glial fibrillary acidic protein
GLI: GLI family zinc finger
GSC: glioma stem cell
GSK3 β: glycogen synthase kinase 3 β
HELLS, helicase: lymphoid specific
HER2: human epidermal growth factor receptor 2
HES5: HES family bHLH transcription factor 5
HEY1: HES-related family bHLH transcription factor with YRPW motif 1
HMG: high motility group
ID1: inhibitor of DNA binding 1 bHLH protein
ID3: inhibitor of DNA binding 3 bHLH protein
IDH1/2: isocitrate dehydrogenase 1/2
JAG: jagged canonical NOTCH ligand
JNK: c-Jun N-terminal kinase
LATS: large tumor suppressor kinase
LEF: lymphoid enhancer binding factor
LRP: LDL receptor-related protein
MAML: mastermind like transcriptional coactivator
MET: mesenchymal-to-epithelial transition
MGMT: O6-methylguanine-DNA methyltransferase
MMP: matrix metalloprotease
MOB1: Mps one binder homolog 1
MST: macrophage stimulating kinase
mTOR: mechanistic target of rapamycin kinase
MYB: MYB proto-oncogene transcription factor
NF1: neurofibromin 1
NF2: neurofibromin 2
NGN: neurogenin bHLH protein
NICD: NOTCH intracellular domain
NKX2.2: NK2 homeobox 2
NMYC: neuroblastoma MYC proto-oncogene bHLH transcription factor
NOTCH: notch receptor
NSC: neural stem cell
OLIG: oligodendrocyte transcription factor
OPC: oligodendrocyte precursor cell
p21: cyclin-dependent kinase inhibitor 1 A
p300: histone acetyltransferase p300
p53: tumor protein 53
PAX: paired box
PDGF: platelet-derived growth factor
PDGFR: platelet-derived growth factor receptor
PI3K: phosphatidylinositol 3-kinase
PML: promyelocytic leukemia
PNS: peripheral nervous system
PRC2: polycomb repressive complex 2
PTCH1: patched homolog 1
PTEN: phosphatase and tensin homolog
RAR: retinoic acid receptor α
RARE, retinoic acid response element: retinoblastoma transcriptional corepressor
RBP4: retinol-binding protein 4
RELA, RELA proto-oncogene: NF-KB subunit
ROBO: roundabout
RXR: retinoid X receptor α
scRNAseq: single cell RNA sequencing
SFRP: secreted frizzled-related protein
SHH: sonic hedgehog
SLIT: slit guidance ligand
SLUG: snail family transcriptional repressor 2
SMAD: small mothers against decapentaplegic homolog
SMARC: SWI/SNF-related, matrix-associated, actin-dependent regulator of chromatin
SMO: smoothened, frizzled class receptor
SNAIL: snail family transcriptional repressor 1
SOX: sex determining region Y (SRY) box
STAT3: signal transducer and activator of transcription 3
SUFU: suppressor of fused homolog
TAZ: tafazzin
TCF: transcription factor
TERT: telomerase reverse transcriptase
TF: transcription factor
TGFRI: TGF β receptor, type I
TGFRII: TGF β receptor, type II
TGFRIII: TGF β receptor, type III
TGF β: transforming growth factor β
TMZ: temozolomide
TP53: tumor protein 53
TSC1/2: tuberous sclerosis complex subunit 1/2
TWIST: twist family bHLH transcription factor 1
VEGF: vascular endothelial growth factor
VEGFR: vascular endothelial growth factor receptor
WNT: wingless-type MMTV integration site family member
YAP: yes-associated protein
ZEB2: zinc finger E-box binding homeobox 2

## References

[B1] Ables J. L. Breunig J. J. Eisch A. J. Rakic P. (2011). Not(ch) just development: notch signalling in the adult brain. *Nat. Rev. Neurosci.* 12 269–283. 10.1038/nrn3024 21505516 PMC3159580

[B2] Acar G. Tanriover G. Demir R. (2012). Angiogenesis in neurological disorders: a review. *Neurol. Res.* 34 627–635. 10.1179/1743132812y.0000000068 22889669

[B3] Ahlfeld J. Favaro R. Pagella P. Kretzschmar H. A. Nicolis S. Schuller U. (2013). Sox2 requirement in sonic hedgehog-associated medulloblastoma. *Cancer Res.* 73 3796–3807. 10.1158/0008-5472.can-13-0238 23596255

[B4] Aldaz P. Otaegi-Ugartemendia M. Saenz-Antonanzas A. Garcia-Puga M. Moreno-Valladares M. Flores J. M. (2020). SOX9 promotes tumor progression through the axis BMI1-p21(CIP). *Sci. Rep.* 10:357. 10.1038/s41598-019-57047-w PMC6962164 31941916

[B5] Alder J. Lee K. J. Jessell T. M. Hatten M. E. (1999). Generation of cerebellar granule neurons in vivo by transplantation of BMP-treated neural progenitor cells. *Nat. Neurosci.* 2 535–540. 10.1038/9189 10448218

[B6] Alonso M. M. Diez-Valle R. Manterola L. Rubio A. Liu D. Cortes-Santiago N. (2011). Genetic and epigenetic modifications of Sox2 contribute to the invasive phenotype of malignant gliomas. *PLoS One* 6:e26740. 10.1371/journal.pone.0026740 22069467 PMC3206066

[B7] Altaba I. R. A. Palma V. Dahmane N. (2002). Hedgehog-Gli signalling and the growth of the brain. *Nat. Rev. Neurosci.* 3 24–33. 11823802 10.1038/nrn704

[B8] Altaba I. R. A. Stecca B. Sanchez P. (2004). Hedgehog–Gli signaling in brain tumors: stem cells and paradevelopmental programs in cancer. *Cancer Lett.* 204 145–157. 10.1016/s0304-3835(03)00451-8 15013214

[B9] Amodeo V. A. D. Betts J. Bartesaghi S. Zhang Y. Richard-Londt A. Ellis M. (2017). A PML/slit axis controls physiological cell migration and cancer invasion in the CNS. *Cell Rep.* 20 411–426. 10.1016/j.celrep.2017.06.047 28700942

[B10] Anderson R. M. Lawrence A. R. Stottmann R. W. Bachiller D. Klingensmith J. (2002). Chordin and noggin promote organizing centers of forebrain development in the mouse. *Development* 129 4975–4987. 12397106 10.1242/dev.129.21.4975

[B11] Annovazzi L. Mellai M. Caldera V. Valente G. Schiffer D. (2011). SOX2 expression and amplification in gliomas and glioma cell lines. *Cancer Genomics Proteomics* 8 139–147. 21518820

[B12] Avilion A. A. Nicolis S. K. Pevny L. H. Perez L. Vivian N. Lovell-Badge R. (2003). Multipotent cell lineages in early mouse development depend on SOX2 function. *Genes Dev.* 17 126–140. 10.1101/gad.224503 12514105 PMC195970

[B13] Ayrault O. Zhao H. Zindy F. Qu C. Sherr C. J. Roussel M. F. (2010). Atoh1 inhibits neuronal differentiation and collaborates with Gli1 to generate medulloblastoma-initiating cells. *Cancer Res.* 70 5618–5627. 10.1158/0008-5472.can-09-3740 20516124 PMC2896438

[B14] Azar S. Leventoux N. Ripoll C. Rigau V. Goze C. Lorcy F. (2018). Cellular and molecular characterization of IDH1-mutated diffuse low grade gliomas reveals tumor heterogeneity and absence of EGFR/PDGFRalpha activation. *Glia* 66 239–255. 10.1002/glia.23240 29027701

[B15] Bachiller D. Klingensmith J. Shneyder N. Tran U. Anderson R. Rossant J. (2003). The role of chordin/Bmp signals in mammalian pharyngeal development and DiGeorge syndrome. *Development* 130 3567–3578. 10.1242/dev.00581 12810603

[B16] Baker S. J. Ellison D. W. Gutmann D. H. (2016). Pediatric gliomas as neurodevelopmental disorders. *Glia* 64 879–895. 26638183 10.1002/glia.22945 PMC4833573

[B17] Ballard M. S. Hinck L. (2012). A roundabout way to cancer. *Adv. Cancer Res.* 114 187–235. 10.1016/b978-0-12-386503-8.00005-3 22588058 PMC4121377

[B18] Balss J. Meyer J. Mueller W. Korshunov A. Hartmann C. Von Deimling A. (2008). Analysis of the IDH1 codon 132 mutation in brain tumors. *Acta Neuropathol.* 116 597–602. 10.1007/s00401-008-0455-2 18985363

[B19] Bannykh S. I. Stolt C. C. Kim J. Perry A. Wegner M. (2006). Oligodendroglial-specific transcriptional factor SOX10 is ubiquitously expressed in human gliomas. *J. Neurooncol.* 76 115–127. 10.1007/s11060-005-5533-x 16205963

[B20] Bao S. Wu Q. Mclendon R. E. Hao Y. Shi Q. Hjelmeland A. B. (2006). Glioma stem cells promote radioresistance by preferential activation of the DNA damage response. *Nature* 444 756–760. 10.1038/nature05236 17051156

[B21] Barcellos-Hoff M. H. (1993). Radiation-induced transforming growth factor beta and subsequent extracellular matrix reorganization in murine mammary gland. *Cancer Res.* 53 3880–3886. 8358713

[B22] Baroti T. Zimmermann Y. Schillinger A. Liu L. Lommes P. Wegner M. (2016). Transcription factors Sox5 and Sox6 exert direct and indirect influences on oligodendroglial migration in spinal cord and forebrain. *Glia* 64 122–138. 10.1002/glia.22919 26345464

[B23] Barry G. Piper M. Lindwall C. Moldrich R. Mason S. Little E. (2008). Specific glial populations regulate hippocampal morphogenesis. *J. Neurosci.* 28 12328–12340. 10.1523/jneurosci.4000-08.2008 19020026 PMC6671696

[B24] Bedell V. M. Yeo S. Y. Park K. W. Chung J. Seth P. Shivalingappa V. (2005). roundabout4 is essential for angiogenesis in vivo. *Proc. Natl. Acad. Sci. U.S.A.* 102 6373–6378. 10.1073/pnas.0408318102 15849270 PMC1088354

[B25] Beier D. Hau P. Proescholdt M. Lohmeier A. Wischhusen J. Oefner P. J. (2007). CD133(+) and CD133(-) glioblastoma-derived cancer stem cells show differential growth characteristics and molecular profiles. *Cancer Res.* 67 4010–4015. 10.1158/0008-5472.can-06-4180 17483311

[B26] Ben-Arie N. Bellen H. J. Armstrong D. L. Mccall A. E. Gordadze P. R. Guo Q. (1997). Math1 is essential for genesis of cerebellar granule neurons. *Nature* 390 169–172. 10.1038/36579 9367153

[B27] Ben-Arie N. Mccall A. E. Berkman S. Eichele G. Bellen H. J. Zoghbi H. Y. (1996). Evolutionary conservation of sequence and expression of the bHLH protein Atonal suggests a conserved role in neurogenesis. *Hum. Mol. Genet.* 5 1207–1216. 10.1093/hmg/5.9.1207 8872459

[B28] Bender S. Tang Y. Lindroth A. M. Hovestadt V. Jones D. T. Kool M. (2013). Reduced H3K27me3 and DNA hypomethylation are major drivers of gene expression in K27M mutant pediatric high-grade gliomas. *Cancer Cell* 24 660–672. 10.1016/j.ccr.2013.10.006 24183680

[B29] Bengoa-Vergniory N. Kypta R. M. (2015). Canonical and noncanonical Wnt signaling in neural stem/progenitor cells. *Cell Mol. Life. Sci.* 72 4157–4172. 10.1007/s00018-015-2028-6 26306936 PMC11113751

[B30] Berezovsky A. D. Poisson L. M. Cherba D. Webb C. P. Transou A. D. Lemke N. W. (2014). Sox2 promotes malignancy in glioblastoma by regulating plasticity and astrocytic differentiation. *Neoplasia* 16 193–206. 10.1016/j.neo.2014.03.006 24726753 PMC4094829

[B31] Berman D. M. Karhadkar S. S. Hallahan A. R. Pritchard J. I. Eberhart C. G. Watkins D. N. (2002). Medulloblastoma growth inhibition by hedgehog pathway blockade. *Science* 297 1559–1561. 10.1126/science.1073733 12202832

[B32] Birch J. L. Coull B. J. Spender L. C. Watt C. Willison A. Syed N. (2020). Multifaceted transforming growth factor-beta (TGFbeta) signalling in glioblastoma. *Cell. Signal.* 72:109638. 10.1016/j.cellsig.2020.109638 32320860

[B33] Blake J. A. Ziman M. R. (2014). Pax genes: regulators of lineage specification and progenitor cell maintenance. *Development* 141 737–751. 10.1242/dev.091785 24496612

[B34] Bleau A. M. Hambardzumyan D. Ozawa T. Fomchenko E. I. Huse J. T. Brennan C. W. (2009). PTEN/PI3K/Akt pathway regulates the side population phenotype and ABCG2 activity in glioma tumor stem-like cells. *Cell Stem Cell* 4 226–235. 10.1016/j.stem.2009.01.007 19265662 PMC3688060

[B35] Bossuyt W. De Geest N. Aerts S. Leenaerts I. Marynen P. Hassan B. A. (2009a). The atonal proneural transcription factor links differentiation and tumor formation in Drosophila. *PLoS Biol.* 7:e40. 10.1371/journal.pbio.1000040 PMC2652389 19243220

[B36] Bossuyt W. Kazanjian A. De Geest N. Van Kelst S. De Hertogh G. Geboes K. (2009b). Atonal homolog 1 is a tumor suppressor gene. *PLoS Biol.* 7:e39. 10.1371/journal.pbio.1000039 PMC2652388 19243219

[B37] Brechbiel J. Miller-Moslin K. Adjei A. A. (2014). Crosstalk between hedgehog and other signaling pathways as a basis for combination therapies in cancer. *Cancer Treat. Rev.* 40 750–759. 10.1016/j.ctrv.2014.02.003 24613036

[B38] Briscoe J. Novitch B. G. (2008). Regulatory pathways linking progenitor patterning, cell fates and neurogenesis in the ventral neural tube. *Philos. Trans. R. Soc. Lond. B Biol. Sci.* 363 57–70. 10.1098/rstb.2006.2012 17282991 PMC2605486

[B39] Bruggeman S. W. Hulsman D. Tanger E. Buckle T. Blom M. Zevenhoven J. (2007). Bmi1 controls tumor development in an Ink4a/Arf-independent manner in a mouse model for glioma. *Cancer Cell* 12 328–341. 10.1016/j.ccr.2007.08.032 17936558

[B40] Buckingham M. Relaix F. (2007). The role of Pax genes in the development of tissues and organs: Pax3 and Pax7 regulate muscle progenitor cell functions. *Annu. Rev. Cell Dev. Biol.* 23 645–673. 10.1146/annurev.cellbio.23.090506.123438 17506689

[B41] Burger M. C. Brucker D. P. Baumgarten P. Ronellenfitsch M. W. Wanka C. Hasselblatt M. (2012). PAX2 is an antiapoptotic molecule with deregulated expression in medulloblastoma. *Int. J. Oncol.* 41 235–241. 22552444 10.3892/ijo.2012.1446

[B42] Bushweller J. H. (2019). Targeting transcription factors in cancer - from undruggable to reality. *Nat. Rev. Cancer* 19 611–624. 10.1038/s41568-019-0196-7 31511663 PMC8820243

[B43] Bylund M. Andersson E. Novitch B. G. Muhr J. (2003). Vertebrate neurogenesis is counteracted by Sox1-3 activity. *Nat. Neurosci.* 6 1162–1168. 10.1038/nn1131 14517545

[B44] Caja L. Bellomo C. Moustakas A. (2015). Transforming growth factor beta and bone morphogenetic protein actions in brain tumors. *FEBS Lett.* 589 1588–1597. 10.1016/j.febslet.2015.04.058 25957771

[B45] Campbell C. E. Piper M. Plachez C. Yeh Y. T. Baizer J. S. Osinski J. M. (2008). The transcription factor Nfix is essential for normal brain development. *BMC Dev. Biol.* 8:52. 10.1186/1471-213x-8-52 18477394 PMC2414869

[B46] Campos B. Weisang S. Osswald F. Ali R. Sedlmeier G. Bageritz J. (2015). Retinoid resistance and multifaceted impairment of retinoic acid synthesis in glioblastoma. *Glia* 63 1850–1859. 10.1002/glia.22849 25944104

[B47] Canazza A. Calatozzolo C. Fumagalli L. Bergantin A. Ghielmetti F. Fariselli L. (2011). Increased migration of a human glioma cell line after in vitro CyberKnife irradiation. *Cancer Biol. Ther.* 12 629–633. 10.4161/cbt.12.7.16862 21775821

[B48] Cau E. Blader P. (2009). Notch activity in the nervous system: to switch or not switch? *Neural Dev.* 4:36. 10.1186/1749-8104-4-36 19799767 PMC2761386

[B49] Cerami E. Gao J. Dogrusoz U. Gross B. E. Sumer S. O. Aksoy B. A. (2012). The cBio cancer genomics portal: an open platform for exploring multidimensional cancer genomics data. *Cancer Discov.* 2 401–404. 10.1158/2159-8290.cd-12-0095 22588877 PMC3956037

[B50] Chang Q. Chen Z. You J. Mcnutt M. A. Zhang T. Han Z. (2007). All-trans-retinoic acid induces cell growth arrest in a human medulloblastoma cell line. *J. Neurooncol.* 84 263–267. 10.1007/s11060-007-9380-9 17453147

[B51] Chaudhry A. Z. Lyons G. E. Gronostajski R. M. (1997). Expression patterns of the four nuclear factor I genes during mouse embryogenesis indicate a potential role in development. *Dev. Dyn.* 208 313–325. 10.1002/(sici)1097-0177(199703)208:3<313::aid-aja3>3.0.co;2-l 9056636

[B52] Chedotal A. (2007). Slits and their receptors. *Adv. Exp. Med. Biol.* 621 65–80. 10.1007/978-0-387-76715-4_5 18269211

[B53] Chen D. Zhao M. Harris S. E. Mi Z. (2004a). Signal transduction and biological functions of bone morphogenetic proteins. *Front. Biosci.* 9 349–358. 10.2741/1090 14766372

[B54] Chen D. Zhao M. Mundy G. R. (2004b). Bone morphogenetic proteins. *Growth Factors* 22 233–241. 15621726 10.1080/08977190412331279890

[B55] Chen J. Kesari S. Rooney C. Strack P. R. Chen J. Shen H. (2010). Inhibition of notch signaling blocks growth of glioblastoma cell lines and tumor neurospheres. *Genes Cancer* 1 822–835. 10.1177/1947601910383564 21127729 PMC2994256

[B56] Chen K. S. Bridges C. R. Lynton Z. Lim J. W. C. Stringer B. W. Rajagopal R. (2020a). Transcription factors NFIA and NFIB induce cellular differentiation in high-grade astrocytoma. *J. Neurooncol.* 146 41–53. 10.1007/s11060-019-03352-3 31760595

[B57] Chen K. S. Lynton Z. Lim J. W. C. Robertson T. Gronostajski R. M. Bunt J. (2020b). NFIA and NFIB function as tumour suppressors in high-grade glioma in mice. *Carcinogenesis* 10.1093/carcin/bgaa139 Online ahead of print. 33346791

[B58] Chen Y. J. Campbell H. G. Wiles A. K. Eccles M. R. Reddel R. R. Braithwaite A. W. (2008). PAX8 regulates telomerase reverse transcriptase and telomerase RNA component in glioma. *Cancer Res.* 68 5724–5732. 10.1158/0008-5472.can-08-0058 18632625

[B59] Cheng S. Y. Yue S. (2008). Role and regulation of human tumor suppressor SUFU in hedgehog signaling. *Adv. Cancer Res.* 101 29–43. 10.1016/s0065-230x(08)00402-8 19055941

[B60] Cheng X. Tan Z. Huang X. Yuan Y. Qin S. Gu Y. (2019). Inhibition of glioma development by ASCL1-mediated direct neuronal reprogramming. *Cells* 8:571. 10.3390/cells8060571 31212628 PMC6627512

[B61] Cheng Y. C. Lee C. J. Badge R. M. Orme A. T. Scotting P. J. (2001). Sox8 gene expression identifies immature glial cells in developing cerebellum and cerebellar tumours. *Brain Res. Mol. Brain Res.* 92 193–200. 10.1016/s0169-328x(01)00147-4 11483257

[B62] Cheung M. Abu-Elmagd M. Clevers H. Scotting P. J. (2000). Roles of Sox4 in central nervous system development. *Brain Res. Mol. Brain Res.* 79 180–191. 10.1016/s0169-328x(00)00109-1 10925158

[B63] Chi N. Epstein J. A. (2002). Getting your pax straight: pax proteins in development and disease. *Trends Genet.* 18 41–47. 10.1016/s0168-9525(01)02594-x 11750700

[B64] Chow L. M. Endersby R. Zhu X. Rankin S. Qu C. Zhang J. (2011). Cooperativity within and among Pten, p53, and Rb pathways induces high-grade astrocytoma in adult brain. *Cancer Cell* 19 305–316. 10.1016/j.ccr.2011.01.039 21397855 PMC3060664

[B65] Connor R. M. Key B. (2002). Expression and role of Roundabout-1 in embryonic Xenopus forebrain. *Dev. Dyn.* 225 22–34. 10.1002/dvdy.10130 12203717

[B66] Corrales J. D. Rocco G. L. Blaess S. Guo Q. Joyner A. L. (2004). Spatial pattern of sonic hedgehog signaling through Gli genes during cerebellum development. *Development* 131 5581–5590. 10.1242/dev.01438 15496441

[B67] Couch J. A. Chen J. Rieff H. I. Uri E. M. Condron B. G. (2004). robo2 and robo3 interact with eagle to regulate serotonergic neuron differentiation. *Development* 131 997–1006. 10.1242/dev.00962 14973268

[B68] Couturier C. P. Ayyadhury S. Le P. U. Nadaf J. Monlong J. Riva G. (2020). Single-cell RNA-seq reveals that glioblastoma recapitulates a normal neurodevelopmental hierarchy. *Nat. Commun.* 11:3406. 10.1038/s41467-020-17186-5 PMC7343844 32641768

[B69] Currie P. D. Ingham P. W. (1996). Induction of a specific muscle cell type by a hedgehog-like protein in zebrafish. *Nature* 382 452–455. 10.1038/382452a0 8684485

[B70] Czapiewski P. Gorczynski A. Radecka K. Wiewiora C. Haybaeck J. Adam P. (2016). Expression of SOX11, PAX5, TTF-1 and ISL-1 in medulloblastoma. *Pathol. Res. Pract.* 212 965–971. 10.1016/j.prp.2016.08.006 27623204

[B71] das Neves L. Duchala C. S. Tolentino-Silva F. Haxhiu M. A. Colmenares C. Macklin W. B. (1999). Disruption of the murine nuclear factor I-A gene (Nfia) results in perinatal lethality, hydrocephalus, and agenesis of the corpus callosum. *Proc. Natl. Acad. Sci. U.S.A.* 96 11946–11951. 10.1073/pnas.96.21.11946 10518556 PMC18392

[B72] Dassule H. R. Lewis P. Bei M. Maas R. Mcmahon A. P. (2000). Sonic hedgehog regulates growth and morphogenesis of the tooth. *Development* 127 4775–4785. 11044393 10.1242/dev.127.22.4775

[B73] de Bont J. M. Kros J. M. Passier M. M. Reddingius R. E. Sillevis Smitt P. A. Luider T. M. (2008). Differential expression and prognostic significance of SOX genes in pediatric medulloblastoma and ependymoma identified by microarray analysis. *Neuro Oncol.* 10 648–660. 10.1215/15228517-2008-032 18577562 PMC2666242

[B74] Delic S. Lottmann N. Stelzl A. Liesenberg F. Wolter M. Gotze S. (2014). MiR-328 promotes glioma cell invasion via SFRP1-dependent Wnt-signaling activation. *Neuro Oncol.* 16 179–190. 10.1093/neuonc/not164 24305703 PMC3895379

[B75] Deneen B. Ho R. Lukaszewicz A. Hochstim C. J. Gronostajski R. M. Anderson D. J. (2006). The transcription factor NFIA controls the onset of gliogenesis in the developing spinal cord. *Neuron* 52 953–968. 10.1016/j.neuron.2006.11.019 17178400

[B76] Dennis D. J. Wilkinson G. Li S. Dixit R. Adnani L. Balakrishnan A. (2017). Neurog2 and Ascl1 together regulate a postmitotic derepression circuit to govern laminar fate specification in the murine neocortex. *Proc. Natl. Acad. Sci. U.S.A.* 114 E4934–E4943. 28584103 10.1073/pnas.1701495114 PMC5488939

[B77] Derynck R. Zhang Y. E. (2003). Smad-dependent and Smad-independent pathways in TGF-beta family signalling. *Nature* 425 577–584. 10.1038/nature02006 14534577

[B78] Desmarais G. Fortin D. Bujold R. Wagner R. Mathieu D. Paquette B. (2012). Infiltration of glioma cells in brain parenchyma stimulated by radiation in the F98/Fischer rat model. *Int. J. Radiat. Biol.* 88 565–574. 10.3109/09553002.2012.692495 22574668

[B79] Di Carlo A. Mariano A. Macchia P. E. Moroni M. C. Beguinot L. Macchia V. (1992). Epidermal growth factor receptor in human brain tumors. *J. Endocrinol. Invest.* 15 31–37. 10.1007/bf03348650 1560188

[B80] Dickinson R. E. Duncan W. C. (2010). The SLIT-ROBO pathway: a regulator of cell function with implications for the reproductive system. *Reproduction* 139 697–704. 10.1530/rep-10-0017 20100881 PMC2971463

[B81] Dixit R. Zimmer C. Waclaw R. R. Mattar P. Shaker T. Kovach C. (2011). Ascl1 participates in Cajal-Retzius cell development in the neocortex. *Cereb. Cortex* 21 2599–2611. 10.1093/cercor/bhr046 21467208

[B82] Dobrotkova V. Chlapek P. Jezova M. Adamkova K. Mazanek P. Sterba J. (2019). Prediction of neuroblastoma cell response to treatment with natural or synthetic retinoids using selected protein biomarkers. *PLoS One* 14:e0218269. 10.1371/journal.pone.0218269 31188873 PMC6561640

[B83] Dong J. Gailani M. R. Pomeroy S. L. Reardon D. Bale A. E. (2000). Identification of PATCHED mutations in medulloblastomas by direct sequencing. *Hum. Mutat.* 16 89–90. 10.1002/1098-1004(200007)16:1<89::aid-humu18>3.0.co;2-7 10874314

[B84] Dressler G. R. (1995). Transcription factors in renal development: the WT1 and Pax-2 story. *Semin. Nephrol.* 15 263–271. 10.1007/978-1-4899-1618-1_21 7569406

[B85] Eccles M. R. (1998). The role of PAX2 in normal and abnormal development of the urinary tract. *Pediatr. Nephrol.* 12 712–720. 10.1007/s004670050533 9874314

[B86] Ehmer U. Sage J. (2016). Control of proliferation and cancer growth by the hippo signaling pathway. *Mol. Cancer Res.* 14 127–140. 10.1158/1541-7786.mcr-15-0305 26432795 PMC4755889

[B87] Erez A. Ilan T. Amariglio N. Muler I. Brok-Simoni F. Rechavi G. (2002). GLI3 is not mutated commonly in sporadic medulloblastomas. *Cancer* 95 28–31. 10.1002/cncr.10642 12115313

[B88] Ericson J. Rashbass P. Schedl A. Brenner-Morton S. Kawakami A. Van Heyningen V. (1997). Pax6 controls progenitor cell identity and neuronal fate in response to graded Shh signaling. *Cell* 90 169–180. 10.1016/s0092-8674(00)80323-2 9230312

[B89] Ferletta M. Uhrbom L. Olofsson T. Ponten F. Westermark B. (2007). Sox10 has a broad expression pattern in gliomas and enhances platelet-derived growth factor-B–induced gliomagenesis. *Mol. Cancer Res.* 5 891–897. 10.1158/1541-7786.mcr-07-0113 17855658

[B90] Fernandes M. Gutin G. Alcorn H. Mcconnell S. K. Hebert J. M. (2007). Mutations in the BMP pathway in mice support the existence of two molecular classes of holoprosencephaly. *Development* 134 3789–3794. 10.1242/dev.004325 17913790

[B91] Fernandez L. A. Northcott P. A. Dalton J. Fraga C. Ellison D. Angers S. (2009). YAP1 is amplified and up-regulated in hedgehog-associated medulloblastomas and mediates Sonic hedgehog-driven neural precursor proliferation. *Genes Dev.* 23 2729–2741. 10.1101/gad.1824509 19952108 PMC2788333

[B92] Filbin M. G. Dabral S. K. Pazyra-Murphy M. F. Ramkissoon S. Kung A. L. Pak E. (2013). Coordinate activation of Shh and PI3K signaling in PTEN-deficient glioblastoma: new therapeutic opportunities. *Nat. Med.* 19 1518–1523. 10.1038/nm.3328 24076665 PMC3923315

[B93] Filbin M. G. Tirosh I. Hovestadt V. Shaw M. L. Escalante L. E. Mathewson N. D. (2018). Developmental and oncogenic programs in H3K27M gliomas dissected by single-cell RNA-seq. *Science* 360 331–335. 29674595 10.1126/science.aao4750 PMC5949869

[B94] Fischer I. Gagner J. P. Law M. Newcomb E. W. Zagzag D. (2005). Angiogenesis in gliomas: biology and molecular pathophysiology. *Brain Pathol.* 15 297–310. 10.1111/j.1750-3639.2005.tb00115.x 16389942 PMC8096031

[B95] Flora A. Klisch T. J. Schuster G. Zoghbi H. Y. (2009). Deletion of Atoh1 disrupts sonic hedgehog signaling in the developing cerebellum and prevents medulloblastoma. *Science* 326 1424–1427. 10.1126/science.1181453 19965762 PMC3638077

[B96] Fogarty M. P. Kessler J. D. Wechsler-Reya R. J. (2005). Morphing into cancer: the role of developmental signaling pathways in brain tumor formation. *J. Neurobiol.* 64 458–475. 10.1002/neu.20166 16041741

[B97] Friedl P. Mayor R. (2017). Tuning collective cell migration by cell-cell junction regulation. *Cold Spring Harb. Perspect. Biol.* 9:a029199. 10.1101/cshperspect.a029199 28096261 PMC5378050

[B98] Fu J. Q. Chen Z. Hu Y. J. Fan Z. H. Guo Z. X. Liang J. Y. (2019). A single factor induces neuronal differentiation to suppress glioma cell growth. *CNS Neurosci. Ther.* 25 486–495. 10.1111/cns.13066 30264483 PMC6488917

[B99] Galli R. Binda E. Orfanelli U. Cipelletti B. Gritti A. De Vitis S. (2004). Isolation and characterization of tumorigenic, stem-like neural precursors from human glioblastoma. *Cancer Res.* 64 7011–7021. 10.1158/0008-5472.can-04-1364 15466194

[B100] Gao J. Aksoy B. A. Dogrusoz U. Dresdner G. Gross B. Sumer S. O. (2013). Integrative analysis of complex cancer genomics and clinical profiles using the cBioPortal. *Sci. Signal.* 6:l1. 10.1126/scisignal.2004088 PMC4160307 23550210

[B101] Gao J. Zhang J. Y. Li Y. H. Ren F. (2015). Decreased expression of SOX9 indicates a better prognosis and inhibits the growth of glioma cells by inducing cell cycle arrest. *Int. J. Clin. Exp. Pathol.* 8 10130–10138. 26617720 PMC4637535

[B102] Gara R. K. Kumari S. Ganju A. Yallapu M. M. Jaggi M. Chauhan S. C. (2015). Slit/Robo pathway: a promising therapeutic target for cancer. *Drug Discov. Today* 20 156–164. 10.1016/j.drudis.2014.09.008 25245168 PMC4445861

[B103] Garcia I. Aldaregia J. Marjanovic Vicentic J. Aldaz P. Moreno-Cugnon L. Torres-Bayona S. (2017). Oncogenic activity of SOX1 in glioblastoma. *Sci. Rep.* 7:46575. 10.1038/srep46575 PMC5397861 28425506

[B104] Gazit R. Krizhanovsky V. Ben-Arie N. (2004). Math1 controls cerebellar granule cell differentiation by regulating multiple components of the Notch signaling pathway. *Development* 131 903–913. 10.1242/dev.00982 14757642

[B105] Glasgow S. M. Carlson J. C. Zhu W. Chaboub L. S. Kang P. Lee H. K. (2017). Glia-specific enhancers and chromatin structure regulate NFIA expression and glioma tumorigenesis. *Nat. Neurosci.* 20 1520–1528. 10.1038/nn.4638 28892058 PMC5919190

[B106] Glasgow S. M. Laug D. Brawley V. S. Zhang Z. Corder A. Yin Z. (2013). The miR-223/nuclear factor I-A axis regulates glial precursor proliferation and tumorigenesis in the CNS. *J. Neurosci.* 33 13560–13568. 10.1523/jneurosci.0321-13.2013 23946414 PMC3742938

[B107] Glasgow S. M. Zhu W. Stolt C. C. Huang T. W. Chen F. Loturco J. J. (2014). Mutual antagonism between Sox10 and NFIA regulates diversification of glial lineages and glioma subtypes. *Nat. Neurosci.* 17 1322–1329. 10.1038/nn.3790 25151262 PMC4313923

[B108] Gnerlich J. L. Ding X. Joyce C. Turner K. Johnson C. D. Chen H. (2019). Increased SOX9 expression in premalignant and malignant pancreatic neoplasms. *Ann. Surg. Oncol.* 26 628–634. 10.1245/s10434-018-6925-4 30357576

[B109] Gojo J. Englinger B. Jiang L. Hubner J. M. Shaw M. L. Hack O. A. (2020). Single-Cell RNA-Seq reveals cellular hierarchies and impaired developmental trajectories in pediatric ependymoma. *Cancer Cell* 38:e49. 10.1016/j.ccell.2020.06.004 PMC7479515 32663469

[B110] Golestaneh N. Mishra B. (2005). TGF-beta, neuronal stem cells and glioblastoma. *Oncogene* 24 5722–5730. 10.1038/sj.onc.1208925 16123805

[B111] Goodrich L. V. Scott M. P. (1998). Hedgehog and patched in neural development and disease. *Neuron* 21 1243–1257. 10.1016/s0896-6273(00)80645-5 9883719

[B112] Graham S. J. Wicher K. B. Jedrusik A. Guo G. Herath W. Robson P. (2014). BMP signalling regulates the pre-implantation development of extra-embryonic cell lineages in the mouse embryo. *Nat. Commun.* 5:5667. 10.1038/ncomms6667 PMC4338527 25514175

[B113] Grausam K. B. Dooyema S. D. R. Bihannic L. Premathilake H. Morrissy A. S. Forget A. (2017). ATOH1 promotes leptomeningeal dissemination and metastasis of sonic hedgehog subgroup medulloblastomas. *Cancer Res.* 77 3766–3777. 10.1158/0008-5472.can-16-1836 28490517 PMC5512702

[B114] Grimmer M. R. Weiss W. A. (2008). BMPs oppose Math1 in cerebellar development and in medulloblastoma. *Genes Dev.* 22 693–699. 10.1101/gad.1657808 18347086 PMC2731664

[B115] Grindley J. C. Davidson D. R. Hill R. E. (1995). The role of Pax-6 in eye and nasal development. *Development* 121 1433–1442. 7789273 10.1242/dev.121.5.1433

[B116] Gronostajski R. M. (2000). Roles of the NFI/CTF gene family in transcription and development. *Gene* 249 31–45. 10.1016/s0378-1119(00)00140-2 10831836

[B117] Groszer M. Erickson R. Scripture-Adams D. D. Lesche R. Trumpp A. Zack J. A. (2001). Negative regulation of neural stem/progenitor cell proliferation by the Pten tumor suppressor gene in vivo. *Science* 294 2186–2189. 10.1126/science.1065518 11691952

[B118] Hallahan A. R. Pritchard J. I. Chandraratna R. A. Ellenbogen R. G. Geyer J. R. Overland R. P. (2003). BMP-2 mediates retinoid-induced apoptosis in medulloblastoma cells through a paracrine effect. *Nat. Med.* 9 1033–1038. 10.1038/nm904 12872164

[B119] Han J. Alvarez-Breckenridge C. A. Wang Q. E. Yu J. (2015). TGF-beta signaling and its targeting for glioma treatment. *Am. J. Cancer Res.* 5 945–955. 26045979 PMC4449428

[B120] Haque A. Banik N. L. Ray S. K. (2007). Emerging role of combination of all-trans retinoic acid and interferon-gamma as chemoimmunotherapy in the management of human glioblastoma. *Neurochem. Res.* 32 2203–2209. 10.1007/s11064-007-9420-z 17676389

[B121] Hatcher A. Yu K. Meyer J. Aiba I. Deneen B. Noebels J. L. (2020). Pathogenesis of peritumoral hyperexcitability in an immunocompetent CRISPR-based glioblastoma model. *J. Clin. Invest.* 130 2286–2300. 10.1172/jci133316 32250339 PMC7190940

[B122] Hayashi Y. Mikawa S. Masumoto K. Katou F. Sato K. (2016). Chordin and noggin expression in the adult rat trigeminal nuclei. *J. Chem. Neuroanat.* 78 36–41. 10.1016/j.jchemneu.2016.08.003 27546891

[B123] Helgager J. Pytel P. Vasudevaraja V. Lee E. Q. Snuderl M. Iorgulescu J. B. (2020). WNT-Activated Medulloblastomas With Hybrid Molecular Subtypes. *JCO Precis. Oncol.* 4:PO.19.00332. 10.1200/PO.19.00332 PMC7446405 32923883

[B124] Helms A. W. Abney A. L. Ben-Arie N. Zoghbi H. Y. Johnson J. E. (2000). Autoregulation and multiple enhancers control Math1 expression in the developing nervous system. *Development* 127 1185–1196. 10683172 10.1242/dev.127.6.1185

[B125] Hemmati H. D. Nakano I. Lazareff J. A. Masterman-Smith M. Geschwind D. H. Bronner-Fraser M. (2003). Cancerous stem cells can arise from pediatric brain tumors. *Proc. Natl. Acad. Sci. U.S.A.* 100 15178–15183. 10.1073/pnas.2036535100 14645703 PMC299944

[B126] Hide T. Takezaki T. Nakatani Y. Nakamura H. Kuratsu J. Kondo T. (2009). Sox11 prevents tumorigenesis of glioma-initiating cells by inducing neuronal differentiation. *Cancer Res.* 69 7953–7959. 10.1158/0008-5472.can-09-2006 19808959

[B127] Hitoshi S. Alexson T. Tropepe V. Donoviel D. Elia A. J. Nye J. S. (2002). Notch pathway molecules are essential for the maintenance, but not the generation, of mammalian neural stem cells. *Genes Dev.* 16 846–858. 10.1101/gad.975202 11937492 PMC186324

[B128] Hopkins C. R. (2016). Inhibitors of the bone morphogenetic protein (BMP) signaling pathway: a patent review (2008-2015). *Expert Opin. Ther. Pat.* 26 1115–1128. 10.1080/13543776.2016.1217330 27476794

[B129] Hornig J. Frob F. Vogl M. R. Hermans-Borgmeyer I. Tamm E. R. Wegner M. (2013). The transcription factors Sox10 and Myrf define an essential regulatory network module in differentiating oligodendrocytes. *PLoS Genet.* 9:e1003907. 10.1371/journal.pgen.1003907 24204311 PMC3814293

[B130] Houillier C. Mokhtari K. Carpentier C. Criniere E. Marie Y. Rousseau A. (2010). Chromosome 9p and 10q losses predict unfavorable outcome in low-grade gliomas. *Neuro Oncol.* 12 2–6. 10.1093/neuonc/nop002 20150361 PMC2940555

[B131] Hover L. D. Abel T. W. Owens P. (2015). Genomic analysis of the BMP family in glioblastomas. *Transl. Oncogenomics* 7 1–9. 10.4137/tog.s22256 25987829 PMC4406393

[B132] Hover L. D. Owens P. Munden A. L. Wang J. Chambless L. B. Hopkins C. R. (2016). Bone morphogenetic protein signaling promotes tumorigenesis in a murine model of high-grade glioma. *Neuro Oncol.* 18 928–938. 10.1093/neuonc/nov310 26683138 PMC4896540

[B133] Hovestadt V. Smith K. S. Bihannic L. Filbin M. G. Shaw M. L. Baumgartner A. (2019). Resolving medulloblastoma cellular architecture by single-cell genomics. *Nature* 572 74–79. 31341285 10.1038/s41586-019-1434-6 PMC6754173

[B134] Hu B. Wang Q. Wang Y. A. Hua S. Sauve C. G. Ong D. (2016). Epigenetic activation of WNT5A drives glioblastoma stem cell differentiation and invasive growth. *Cell* 167 1281–1295 e1218. 27863244 10.1016/j.cell.2016.10.039 PMC5320931

[B135] Hu J. G. Lu H. Z. Wang Y. X. Bao M. S. Zhao B. M. Zhou J. S. (2010). BMP signaling mediates astrocyte differentiation of oligodendrocyte progenitor cells. *Tohoku J. Exp. Med.* 222 195–200. 10.1620/tjem.222.195 21041993

[B136] Huber M. A. Kraut N. Beug H. (2005). Molecular requirements for epithelial-mesenchymal transition during tumor progression. *Curr. Opin. Cell Biol.* 17 548–558. 10.1016/j.ceb.2005.08.001 16098727

[B137] Ichimura K. Pearson D. M. Kocialkowski S. Backlund L. M. Chan R. Jones D. T. (2009). IDH1 mutations are present in the majority of common adult gliomas but rare in primary glioblastomas. *Neuro Oncol.* 11 341–347. 10.1215/15228517-2009-025 19435942 PMC2743214

[B138] Idbaih A. Kouwenhoven M. Jeuken J. Carpentier C. Gorlia T. Kros J. M. (2008). Chromosome 1p loss evaluation in anaplastic oligodendrogliomas. *Neuropathology* 28 440–443. 10.1111/j.1440-1789.2008.00863.x 18312547

[B139] Ikushima H. Todo T. Ino Y. Takahashi M. Miyazawa K. Miyazono K. (2009). Autocrine TGF-beta signaling maintains tumorigenicity of glioma-initiating cells through Sry-related HMG-box factors. *Cell Stem Cell* 5 504–514. 10.1016/j.stem.2009.08.018 19896441

[B140] Ikushima H. Todo T. Ino Y. Takahashi M. Saito N. Miyazawa K. (2011). Glioma-initiating cells retain their tumorigenicity through integration of the Sox axis and Oct4 protein. *J. Biol. Chem.* 286 41434–41441. 10.1074/jbc.m111.300863 21987575 PMC3308855

[B141] Ingham P. W. (1998). Transducing hedgehog: the story so far. *EMBO J.* 17 3505–3511. 10.1093/emboj/17.13.3505 9649421 PMC1170687

[B142] Jablonska B. Aguirre A. Raymond M. Szabo G. Kitabatake Y. Sailor K. A. (2010). Chordin-induced lineage plasticity of adult SVZ neuroblasts after demyelination. *Nat. Neurosci.* 13 541–550. 10.1038/nn.2536 20418875 PMC4059417

[B143] Jain R. K. Di Tomaso E. Duda D. G. Loeffler J. S. Sorensen A. G. Batchelor T. T. (2007). Angiogenesis in brain tumours. *Nat. Rev. Neurosci.* 8 610–622. 17643088 10.1038/nrn2175

[B144] Jermyn M. Desroches J. Mercier J. St-Arnaud K. Guiot M. C. Leblond F. (2016). Raman spectroscopy detects distant invasive brain cancer cells centimeters beyond MRI capability in humans. *Biomed. Opt. Express* 7 5129–5137. 10.1364/boe.7.005129 28018730 PMC5175557

[B145] Jin X. Jeon H. Y. Joo K. M. Kim J. K. Jin J. Kim S. H. (2011). Frizzled 4 regulates stemness and invasiveness of migrating glioma cells established by serial intracranial transplantation. *Cancer Res.* 71 3066–3075. 10.1158/0008-5472.can-10-1495 21363911

[B146] Jin X. Jin X. Jung J. E. Beck S. Kim H. (2013). Cell surface Nestin is a biomarker for glioma stem cells. *Biochem. Biophys. Res. Commun.* 433 496–501. 10.1016/j.bbrc.2013.03.021 23524267

[B147] John Lin C. C. Yu K. Hatcher A. Huang T. W. Lee H. K. Carlson J. (2017). Identification of diverse astrocyte populations and their malignant analogs. *Nat. Neurosci.* 20 396–405. 10.1038/nn.4493 28166219 PMC5824716

[B148] Joo K. M. Kim S. Y. Jin X. Song S. Y. Kong D. S. Lee J. I. (2008). Clinical and biological implications of CD133-positive and CD133-negative cells in glioblastomas. *Lab. Invest.* 88 808–815. 10.1038/labinvest.2008.57 18560366

[B149] Jordan C. T. Guzman M. L. Noble M. (2006). Cancer stem cells. *N. Engl. J. Med.* 355 1253–1261. 16990388 10.1056/NEJMra061808

[B150] Juratli T. A. Schackert G. Krex D. (2013). Current status of local therapy in malignant gliomas–a clinical review of three selected approaches. *Pharmacol. Ther.* 139 341–358. 10.1016/j.pharmthera.2013.05.003 23694764

[B151] Kadin M. E. Rubinstein L. J. Nelson J. S. (1970). Neonatal cerebellar medulloblastoma originating from the fetal external granular layer. *J. Neuropathol. Exp. Neurol.* 29 583–600. 10.1097/00005072-197010000-00005 5471923

[B152] Kamachi Y. Kondoh H. (2013). Sox proteins: regulators of cell fate specification and differentiation. *Development* 140 4129–4144. 10.1242/dev.091793 24086078

[B153] Kamino M. Kishida M. Kibe T. Ikoma K. Iijima M. Hirano H. (2011). Wnt-5a signaling is correlated with infiltrative activity in human glioma by inducing cellular migration and MMP-2. *Cancer Sci.* 102 540–548. 10.1111/j.1349-7006.2010.01815.x 21205070

[B154] Kaminska B. Cyranowski S. (2020). Recent advances in understanding mechanisms of TGF beta signaling and its role in glioma pathogenesis. *Adv. Exp. Med. Biol.* 1202 179–201. 10.1007/978-3-030-30651-9_9 32034714

[B155] Kaminska B. Kocyk M. Kijewska M. (2013). TGF beta signaling and its role in glioma pathogenesis. *Adv. Exp. Med. Biol.* 986 171–187. 10.1007/978-94-007-4719-7_9 22879069

[B156] Kiecker C. Niehrs C. (2001). A morphogen gradient of Wnt/beta-catenin signalling regulates anteroposterior neural patterning in Xenopus. *Development* 128 4189–4201. 11684656 10.1242/dev.128.21.4189

[B157] Kishigami S. Mishina Y. (2005). BMP signaling and early embryonic patterning. *Cytokine Growth Factor Rev.* 16 265–278. 10.1016/j.cytogfr.2005.04.002 15871922

[B158] Klisch T. J. Vainshtein A. Patel A. J. Zoghbi H. Y. (2017). Jak2-mediated phosphorylation of Atoh1 is critical for medulloblastoma growth. *Elife* 6:e31181. 10.7554/eLife.31181 PMC5736349 29168692

[B159] Klose A. Waerzeggers Y. Monfared P. Vukicevic S. Kaijzel E. L. Winkeler A. (2011). Imaging bone morphogenetic protein 7 induced cell cycle arrest in experimental gliomas. *Neoplasia* 13 276–285. 10.1593/neo.101540 21390190 PMC3050870

[B160] Koch U. Radtke F. (2007). Notch and cancer: a double-edged sword. *Cell Mol. Life Sci.* 64 2746–2762. 10.1007/s00018-007-7164-1 17687513 PMC11136344

[B161] Komiya Y. Habas R. (2008). Wnt signal transduction pathways. *Organogenesis* 4 68–75. 10.4161/org.4.2.5851 19279717 PMC2634250

[B162] Kondo T. Raff M. (2000). Basic helix-loop-helix proteins and the timing of oligodendrocyte differentiation. *Development* 127 2989–2998. 10862737 10.1242/dev.127.14.2989

[B163] Kongkham P. N. Northcott P. A. Croul S. E. Smith C. A. Taylor M. D. Rutka J. T. (2010). The SFRP family of WNT inhibitors function as novel tumor suppressor genes epigenetically silenced in medulloblastoma. *Oncogene* 29 3017–3024. 10.1038/onc.2010.32 20208569

[B164] Kool M. Koster J. Bunt J. Hasselt N. E. Lakeman A. Van Sluis P. (2008). Integrated genomics identifies five medulloblastoma subtypes with distinct genetic profiles, pathway signatures and clinicopathological features. *PLoS One* 3:e3088. 10.1371/journal.pone.0003088 18769486 PMC2518524

[B165] Korkolopoulou P. Levidou G. El-Habr E. A. Adamopoulos C. Fragkou P. Boviatsis E. (2013). Sox11 expression in astrocytic gliomas: correlation with nestin/c-Met/IDH1-R132H expression phenotypes, p-Stat-3 and survival. *Br. J. Cancer* 108 2142–2152. 10.1038/bjc.2013.176 23619925 PMC3670505

[B166] Kozmik Z. Sure U. Ruedi D. Busslinger M. Aguzzi A. (1995). Deregulated expression of PAX5 in medulloblastoma. *Proc. Natl. Acad. Sci. U.S.A.* 92 5709–5713. 10.1073/pnas.92.12.5709 7777574 PMC41766

[B167] Kristoffersen K. Villingshoj M. Poulsen H. S. Stockhausen M. T. (2013). Level of Notch activation determines the effect on growth and stem cell-like features in glioblastoma multiforme neurosphere cultures. *Cancer Biol. Ther.* 14 625–637. 10.4161/cbt.24595 23792644 PMC3742492

[B168] Lai H. C. Seal R. P. Johnson J. E. (2016). Making sense out of spinal cord somatosensory development. *Development* 143 3434–3448. 10.1242/dev.139592 27702783 PMC5087618

[B169] Lang D. Powell S. K. Plummer R. S. Young K. P. Ruggeri B. A. (2007). PAX genes: roles in development, pathophysiology, and cancer. *Biochem. Pharmacol.* 73 1–14. 10.1016/j.bcp.2006.06.024 16904651

[B170] Larrain J. Bachiller D. Lu B. Agius E. Piccolo S. De Robertis E. M. (2000). BMP-binding modules in chordin: a model for signalling regulation in the extracellular space. *Development* 127 821–830. 10648240 10.1242/dev.127.4.821 PMC2280033

[B171] Lasky J. L. Wu H. (2005). Notch signaling, brain development, and human disease. *Pediatr. Res.* 57 104R–109R. 10.1203/01.PDR.0000159632.70510.3D 15817497

[B172] Lathia J. D. Gallagher J. Heddleston J. M. Wang J. Eyler C. E. Macswords J. (2010). Integrin alpha 6 regulates glioblastoma stem cells. *Cell Stem Cell* 6 421–432. 10.1016/j.stem.2010.02.018 20452317 PMC2884275

[B173] Lee C. J. Appleby V. J. Orme A. T. Chan W. I. Scotting P. J. (2002). Differential expression of SOX4 and SOX11 in medulloblastoma. *J. Neurooncol.* 57 201–214. 12125983 10.1023/a:1015773818302

[B174] Lee J. Kotliarova S. Kotliarov Y. Li A. Su Q. Donin N. M. (2006). Tumor stem cells derived from glioblastomas cultured in bFGF and EGF more closely mirror the phenotype and genotype of primary tumors than do serum-cultured cell lines. *Cancer Cell* 9 391–403. 10.1016/j.ccr.2006.03.030 16697959

[B175] Lee Y. Lee J. K. Ahn S. H. Lee J. Nam D. H. (2016). WNT signaling in glioblastoma and therapeutic opportunities. *Lab. Invest.* 96 137–150. 10.1038/labinvest.2015.140 26641068

[B176] Lewis K. E. Eisen J. S. (2001). Hedgehog signaling is required for primary motoneuron induction in zebrafish. *Development* 128 3485–3495. 11566854 10.1242/dev.128.18.3485

[B177] Li H. S. Chen J. H. Wu W. Fagaly T. Zhou L. Yuan W. (1999). Vertebrate slit, a secreted ligand for the transmembrane protein roundabout, is a repellent for olfactory bulb axons. *Cell* 96 807–818. 10.1016/s0092-8674(00)80591-7 10102269

[B178] Li L. Li Q. Chen X. Xu M. Li X. Nie L. (2015). SOX4 is overexpressed in diffusely infiltrating astrocytoma and confers poor prognosis. *Neuropathology* 35 510–517. 10.1111/neup.12212 26096696

[B179] Liao W. X. Wing D. A. Geng J. G. Chen D. B. (2010). Perspectives of SLIT/ROBO signaling in placental angiogenesis. *Histol. Histopathol.* 25 1181–1190. 20607660 10.14670/HH-25.1181 PMC8900672

[B180] Ligon K. L. Alberta J. A. Kho A. T. Weiss J. Kwaan M. R. Nutt C. L. (2004). The oligodendroglial lineage marker OLIG2 is universally expressed in diffuse gliomas. *J. Neuropathol. Exp. Neurol.* 63 499–509. 10.1093/jnen/63.5.499 15198128

[B181] Ligon K. L. Huillard E. Mehta S. Kesari S. Liu H. Alberta J. A. (2007). Olig2-regulated lineage-restricted pathway controls replication competence in neural stem cells and malignant glioma. *Neuron* 53 503–517. 10.1016/j.neuron.2007.01.009 17296553 PMC1810344

[B182] Liu H. Liu Z. Jiang B. Peng R. Ma Z. Lu J. (2015). SOX9 overexpression promotes glioma metastasis via Wnt/beta-CATENIN SIGNALING. *Cell Biochem. Biophys.* 73 205–212. 10.1007/s12013-015-0647-z 25716338

[B183] Liu Y. C. Wang Y. Z. (2015). Role of Yes-associated protein 1 in gliomas: pathologic and therapeutic aspects. *Tumour Biol.* 36 2223–2227. 10.1007/s13277-015-3297-2 25750037

[B184] Louis D. N. Perry A. Reifenberger G. Von Deimling A. Figarella-Branger D. Cavenee W. K. (2016). The 2016 world health organization classification of tumors of the central nervous system: a summary. *Acta Neuropathol.* 131 803–820. 10.1007/s00401-016-1545-1 27157931

[B185] Lu F. Chen Y. Zhao C. Wang H. He D. Xu L. (2016). Olig2-dependent reciprocal shift in PDGF and EGF receptor signaling regulates tumor phenotype and mitotic growth in malignant glioma. *Cancer Cell* 29 669–683. 10.1016/j.ccell.2016.03.027 27165742 PMC4946168

[B186] Lu Q. R. Park J. K. Noll E. Chan J. A. Alberta J. Yuk D. (2001). Oligodendrocyte lineage genes (OLIG) as molecular markers for human glial brain tumors. *Proc. Natl. Acad. Sci. U.S.A.* 98 10851–10856. 10.1073/pnas.181340798 11526205 PMC58563

[B187] Luo K. (2017). Signaling cross talk between TGF-beta/Smad and other signaling pathways. *Cold Spring Harb. Perspect. Biol.* 9:a022137. 10.1101/cshperspect.a022137 27836834 PMC5204325

[B188] Machein M. R. Plate K. H. (2000). VEGF in brain tumors. *J. Neurooncol.* 50 109–120. 11245271 10.1023/a:1006416003964

[B189] Maden M. (2007). Retinoic acid in the development, regeneration and maintenance of the nervous system. *Nat. Rev. Neurosci.* 8 755–765. 10.1038/nrn2212 17882253

[B190] Manoranjan B. Venugopal C. Mcfarlane N. Doble B. W. Dunn S. E. Scheinemann K. (2012). Medulloblastoma stem cells: where development and cancer cross pathways. *Pediatr. Res.* 71 516–522. 10.1038/pr.2011.62 22430388

[B191] Mansouri A. Goudreau G. Gruss P. (1999). Pax genes and their role in organogenesis. *Cancer Res.* 59 1707–1709. 10197584

[B192] Mansouri S. Nejad R. Karabork M. Ekinci C. Solaroglu I. Aldape K. D. (2016). Sox2: regulation of expression and contribution to brain tumors. *CNS Oncol.* 5 159–173. 10.2217/cns-2016-0001 27230973 PMC6042636

[B193] Marino S. (2005). Medulloblastoma: developmental mechanisms out of control. *Trends Mol. Med.* 11 17–22. 10.1016/j.molmed.2004.11.008 15649818

[B194] Marjanovic Vicentic J. Drakulic D. Garcia I. Vukovic V. Aldaz P. Puskas N. (2019). SOX3 can promote the malignant behavior of glioblastoma cells. *Cell Oncol.* 42 41–54. 10.1007/s13402-018-0405-5 30209685 PMC12994348

[B195] Mason S. Piper M. Gronostajski R. M. Richards L. J. (2009). Nuclear factor one transcription factors in CNS development. *Mol. Neurobiol.* 39 10–23. 10.1007/s12035-008-8048-6 19058033

[B196] Mellai M. Monzeglio O. Piazzi A. Caldera V. Annovazzi L. Cassoni P. (2012). MGMT promoter hypermethylation and its associations with genetic alterations in a series of 350 brain tumors. *J. Neurooncol.* 107 617–631. 10.1007/s11060-011-0787-y 22287028

[B197] Mertsch S. Schmitz N. Jeibmann A. Geng J. G. Paulus W. Senner V. (2008). Slit2 involvement in glioma cell migration is mediated by Robo1 receptor. *J. Neurooncol.* 87 1–7. 10.1007/s11060-007-9484-2 17968499

[B198] Merve A. Dubuc A. M. Zhang X. Remke M. Baxter P. A. Li X. N. (2014). Polycomb group gene BMI1 controls invasion of medulloblastoma cells and inhibits BMP-regulated cell adhesion. *Acta Neuropathol. Commun.* 2:10. 10.1038/scibx.2011.10 PMC3928978 24460684

[B199] Mikawa S. Sato K. (2014). Chordin expression in the adult rat brain. *Neuroscience* 258 16–33. 10.1016/j.neuroscience.2013.11.006 24231736

[B200] Moscoso L. M. Sanes J. R. (1995). Expression of four immunoglobulin superfamily adhesion molecules (L1, Nr-CAM/Bravo, neurofascin/ABGP, and N-CAM) in the developing mouse spinal cord. *J. Comp. Neurol.* 352 321–334. 10.1002/cne.903520302 7706555

[B201] Mulligan K. A. Cheyette B. N. (2012). Wnt signaling in vertebrate neural development and function. *J. Neuroimmune Pharmacol.* 7 774–787. 10.1007/s11481-012-9404-x 23015196 PMC3518582

[B202] Muratovska A. Zhou C. He S. Goodyer P. Eccles M. R. (2003). Paired-Box genes are frequently expressed in cancer and often required for cancer cell survival. *Oncogene* 22 7989–7997. 10.1038/sj.onc.1206766 12970747

[B203] Nakatani H. Martin E. Hassani H. Clavairoly A. Maire C. L. Viadieu A. (2013). Ascl1/Mash1 promotes brain oligodendrogenesis during myelination and remyelination. *J. Neurosci.* 33 9752–9768. 10.1523/jneurosci.0805-13.2013 23739972 PMC3892435

[B204] Nanni L. Ming J. E. Bocian M. Steinhaus K. Bianchi D. W. Die-Smulders C. (1999). The mutational spectrum of the sonic hedgehog gene in holoprosencephaly: SHH mutations cause a significant proportion of autosomal dominant holoprosencephaly. *Hum. Mol. Genet.* 8 2479–2488. 10.1093/hmg/8.13.2479 10556296

[B205] Neftel C. Laffy J. Filbin M. G. Hara T. Shore M. E. Rahme G. J. (2019). An integrative model of cellular states. plasticity, and genetics for glioblastoma. *Cell* 178 835–849 e821. 31327527 10.1016/j.cell.2019.06.024 PMC6703186

[B206] Nelms B. L. Labosky P. A. (2010). *Transcriptional Control of Neural Crest Development.* San Rafael, CA: Biota Publishing. 21452438

[B207] Neve A. Migliavacca J. Capdeville C. Schonholzer M. T. Gries A. Ma M. (2019). Crosstalk between SHH and FGFR signaling pathways controls tissue invasion in medulloblastoma. *Cancers* 11:1985. 10.3390/cancers11121985 31835472 PMC6966681

[B208] Nevo I. Woolard K. Cam M. Li A. Webster J. D. Kotliarov Y. (2014). Identification of molecular pathways facilitating glioma cell invasion in situ. *PLoS One* 9:e111783. 10.1371/journal.pone.0111783 25365423 PMC4218815

[B209] Nguemgo Kouam P. Rezniczek G. A. Kochanneck A. Priesch-Grzeszkowiak B. Hero T. Adamietz I. A. (2018). Robo1 and vimentin regulate radiation-induced motility of human glioblastoma cells. *PLoS One* 13:e0198508. 10.1371/journal.pone.0198508 29864155 PMC5986140

[B210] Nicot C. (2015). Tumor suppressor inactivation in the pathogenesis of adult T-Cell leukemia. *J. Oncol.* 2015:183590. 10.1155/2015/183590 PMC4478360 26170835

[B211] Nieto M. Schuurmans C. Britz O. Guillemot F. (2001). Neural bHLH genes control the neuronal versus glial fate decision in cortical progenitors. *Neuron* 29 401–413. 10.1016/s0896-6273(01)00214-8 11239431

[B212] Nikolopoulou E. Galea G. L. Rolo A. Greene N. D. Copp A. J. (2017). Neural tube closure: cellular, molecular and biomechanical mechanisms. *Development* 144 552–566. 10.1242/dev.145904 28196803 PMC5325323

[B213] Northcott P. A. Korshunov A. Witt H. Hielscher T. Eberhart C. G. Mack S. (2011). Medulloblastoma comprises four distinct molecular variants. *J. Clin. Oncol.* 29 1408–1414. 10.1200/jco.2009.27.4324 20823417 PMC4874239

[B214] Oasa S. Vukojevic V. Rigler R. Tsigelny I. F. Changeux J. P. Terenius L. (2020). A strategy for designing allosteric modulators of transcription factor dimerization. *Proc. Natl. Acad. Sci. U.S.A.* 117 2683–2686. 10.1073/pnas.1915531117 31953259 PMC7007557

[B215] Oosterveen T. Kurdija S. Alekseenko Z. Uhde C. W. Bergsland M. Sandberg M. (2012). Mechanistic differences in the transcriptional interpretation of local and long-range Shh morphogen signaling. *Dev. Cell* 23 1006–1019. 10.1016/j.devcel.2012.09.015 23153497

[B216] Ostrom Q. T. Gittleman H. Xu J. Kromer C. Wolinsky Y. Kruchko C. (2016). CBTRUS statistical report: primary brain and other central nervous system tumors diagnosed in the United States in 2009-2013. *Neuro Oncol.* 18 v1–v75. 28475809 10.1093/neuonc/now207 PMC8483569

[B217] Ozdinler P. H. Erzurumlu R. S. (2002). Slit2, a branching-arborization factor for sensory axons in the mammalian CNS. *J. Neurosci.* 22 4540–4549. 10.1523/jneurosci.22-11-04540.2002 12040061 PMC4260804

[B218] Pacary E. Heng J. Azzarelli R. Riou P. Castro D. Lebel-Potter M. (2011). Proneural transcription factors regulate different steps of cortical neuron migration through Rnd-mediated inhibition of RhoA signaling. *Neuron* 69 1069–1084. 10.1016/j.neuron.2011.02.018 21435554 PMC3383999

[B219] Park N. I. Guilhamon P. Desai K. Mcadam R. F. Langille E. O’connor M. (2017). ASCL1 reorganizes chromatin to direct neuronal fate and suppress tumorigenicity of glioblastoma stem cells. *Cell Stem Cell* 21 209–224 e207. 28712938 10.1016/j.stem.2017.06.004

[B220] Parsons D. W. Jones S. Zhang X. Lin J. C. Leary R. J. Angenendt P. (2008). An integrated genomic analysis of human glioblastoma multiforme. *Science* 321 1807–1812. 18772396 10.1126/science.1164382 PMC2820389

[B221] Patel A. P. Tirosh I. Trombetta J. J. Shalek A. K. Gillespie S. M. Wakimoto H. (2014). Single-cell RNA-seq highlights intratumoral heterogeneity in primary glioblastoma. *Science* 344 1396–1401. 10.1126/science.1254257 24925914 PMC4123637

[B222] Pearson J. R. D. Regad T. (2017). Targeting cellular pathways in glioblastoma multiforme. *Signal. Transduct. Target. Ther.* 2:17040. 10.1038/sigtrans.2017.40 PMC5661637 29263927

[B223] Peiris P. M. Abramowski A. Mcginnity J. Doolittle E. Toy R. Gopalakrishnan R. (2015). Treatment of invasive brain tumors using a chain-like nanoparticle. *Cancer Res.* 75 1356–1365. 10.1158/0008-5472.can-14-1540 25627979 PMC4383708

[B224] Pelullo M. Zema S. Nardozza F. Checquolo S. Screpanti I. Bellavia D. (2019). Wnt, notch, and TGF-beta pathways impinge on hedgehog signaling complexity: an open window on cancer. *Front. Genet.* 10:711. 10.3389/fgene.2019.00711 PMC6736567 31552081

[B225] Penuelas S. Anido J. Prieto-Sanchez R. M. Folch G. Barba I. Cuartas I. (2009). TGF-beta increases glioma-initiating cell self-renewal through the induction of LIF in human glioblastoma. *Cancer Cell* 15 315–327. 10.1016/j.ccr.2009.02.011 19345330

[B226] Perla A. Fratini L. Cardoso P. S. Nor C. Brunetto A. T. Brunetto A. L. (2020). Histone deacetylase inhibitors in pediatric brain cancers: biological activities and therapeutic potential. *Front. Cell Dev. Biol.* 8:546. 10.3389/fcell.2020.00546 PMC7365945 32754588

[B227] Peterson K. A. Nishi Y. Ma W. Vedenko A. Shokri L. Zhang X. (2012). Neural-specific Sox2 input and differential Gli-binding affinity provide context and positional information in Shh-directed neural patterning. *Genes Dev.* 26 2802–2816. 10.1101/gad.207142.112 23249739 PMC3533082

[B228] Phi J. H. Kim J. H. Eun K. M. Wang K. C. Park K. H. Choi S. A. (2010). Upregulation of SOX2, NOTCH1, and ID1 in supratentorial primitive neuroectodermal tumors: a distinct differentiation pattern from that of medulloblastomas. *J. Neurosurg. Pediatr.* 5 608–614. 10.3171/2010.2.peds1065 20515335

[B229] Phi J. H. Park S. H. Kim S. K. Paek S. H. Kim J. H. Lee Y. J. (2008). Sox2 expression in brain tumors: a reflection of the neuroglial differentiation pathway. *Am. J. Surg. Pathol.* 32 103–112. 10.1097/pas.0b013e31812f6ba6 18162777

[B230] Phillips H. S. Kharbanda S. Chen R. Forrest W. F. Soriano R. H. Wu T. D. (2006). Molecular subclasses of high-grade glioma predict prognosis, delineate a pattern of disease progression, and resemble stages in neurogenesis. *Cancer Cell* 9 157–173. 10.1016/j.ccr.2006.02.019 16530701

[B231] Piccirillo S. G. Vescovi A. L. (2006). Bone morphogenetic proteins regulate tumorigenicity in human glioblastoma stem cells. *Ernst Schering Found. Symp. Proc.* 5 59–81. 10.1007/2789_2007_044 17939295

[B232] Pietsch T. Waha A. Koch A. Kraus J. Albrecht S. Tonn J. (1997). Medulloblastomas of the desmoplastic variant carry mutations of the human homologue of Drosophila patched. *Cancer Res.* 57 2085–2088. 9187099

[B233] Platten M. Wick W. Wild-Bode C. Aulwurm S. Dichgans J. Weller M. (2000). Transforming growth factors beta(1) (TGF-beta(1)) and TGF-beta(2) promote glioma cell migration via Up-regulation of alpha(V)beta(3) integrin expression. *Biochem. Biophys. Res. Commun.* 268 607–611. 10679251 10.1006/bbrc.2000.2176

[B234] Plouffe S. W. Hong A. W. Guan K. L. (2015). Disease implications of the Hippo/YAP pathway. *Trends Mol. Med.* 21 212–222. 10.1016/j.molmed.2015.01.003 25702974 PMC4385444

[B235] Po A. Ferretti E. Miele E. De Smaele E. Paganelli A. Canettieri G. (2010). Hedgehog controls neural stem cells through p53-independent regulation of Nanog. *EMBO J.* 29 2646–2658. 10.1038/emboj.2010.131 20581804 PMC2928686

[B236] Prager B. C. Bhargava S. Mahadev V. Hubert C. G. Rich J. N. (2020). Glioblastoma stem cells: driving resilience through chaos. *Trends Cancer* 6 223–235. 10.1016/j.trecan.2020.01.009 32101725 PMC8779821

[B237] Purow B. W. Sundaresan T. K. Burdick M. J. Kefas B. A. Comeau L. D. Hawkinson M. P. (2008). Notch-1 regulates transcription of the epidermal growth factor receptor through p53. *Carcinogenesis* 29 918–925. 10.1093/carcin/bgn079 18359760 PMC2902388

[B238] Qin F. Zhang H. Ma L. Liu X. Dai K. Li W. (2015). Low expression of Slit2 and Robo1 is associated with poor prognosis and brain-specific metastasis of breast cancer patients. *Sci. Rep.* 5:14430. 10.1038/srep14430 PMC4585856 26400100

[B239] Raffel C. Jenkins R. B. Frederick L. Hebrink D. Alderete B. Fults D. W. (1997). Sporadic medulloblastomas contain PTCH mutations. *Cancer Res.* 57 842–845. 9041183

[B240] Reddi A. H. (2001). Interplay between bone morphogenetic proteins and cognate binding proteins in bone and cartilage development: noggin, chordin and DAN. *Arthritis Res.* 3 1–5. 10.1007/978-3-0348-7857-9_1 11178121 PMC128877

[B241] Reitman Z. J. Yan H. (2010). Isocitrate dehydrogenase 1 and 2 mutations in cancer: alterations at a crossroads of cellular metabolism. *J. Natl. Cancer Inst.* 102 932–941. 10.1093/jnci/djq187 20513808 PMC2897878

[B242] Reynolds C. P. Matthay K. K. Villablanca J. G. Maurer B. J. (2003). Retinoid therapy of high-risk neuroblastoma. *Cancer Lett.* 197 185–192. 10.1016/s0304-3835(03)00108-3 12880980

[B243] Rheinbay E. Suva M. L. Gillespie S. M. Wakimoto H. Patel A. P. Shahid M. (2013). An aberrant transcription factor network essential for Wnt signaling and stem cell maintenance in glioblastoma. *Cell Rep.* 3 1567–1579. 10.1016/j.celrep.2013.04.021 23707066 PMC3774301

[B244] Robinson M. H. Maximov V. Lallani S. Farooq H. Taylor M. D. Read R. D. (2019). Upregulation of the chromatin remodeler HELLS is mediated by YAP1 in sonic hedgehog medulloblastoma. *Sci. Rep.* 9:13611. 10.1038/s41598-019-50088-1 PMC6754407 31541170

[B245] Ronca F. Andersen J. S. Paech V. Margolis R. U. (2001). Characterization of Slit protein interactions with glypican-1. *J. Biol. Chem.* 276 29141–29147. 10.1074/jbc.m100240200 11375980

[B246] Rooprai H. K. Rucklidge G. J. Panou C. Pilkington G. J. (2000). The effects of exogenous growth factors on matrix metalloproteinase secretion by human brain tumour cells. *Br. J. Cancer* 82 52–55. 10.1054/bjoc.1999.0876 10638966 PMC2363180

[B247] Rose M. F. Ren J. Ahmad K. A. Chao H. T. Klisch T. J. Flora A. (2009). Math1 is essential for the development of hindbrain neurons critical for perinatal breathing. *Neuron* 64 341–354. 10.1016/j.neuron.2009.10.023 19914183 PMC2818435

[B248] Ross S. A. Mccaffery P. J. Drager U. C. De Luca L. M. (2000). Retinoids in embryonal development. *Physiol. Rev.* 80 1021–1054. 10.1152/physrev.2000.80.3.1021 10893430

[B249] Rousseau A. Nutt C. L. Betensky R. A. Iafrate A. J. Han M. Ligon K. L. (2006). Expression of oligodendroglial and astrocytic lineage markers in diffuse gliomas: use of YKL-40, ApoE, ASCL1, and NKX2-2. *J. Neuropathol. Exp. Neurol.* 65 1149–1156. 10.1097/01.jnen.0000248543.90304.2b 17146289

[B250] Rowitch D. H. S-Jacques B. Lee S. M. Flax J. D. Snyder E. Y. Mcmahon A. P. (1999). Sonic hedgehog regulates proliferation and inhibits differentiation of CNS precursor cells. *J. Neurosci.* 19 8954–8965. 10.1523/jneurosci.19-20-08954.1999 10516314 PMC6782773

[B251] Rubenstein J. L. (2011). Annual research review: development of the cerebral cortex: implications for neurodevelopmental disorders. *J. Child Psychol. Psychiatry* 52 339–355. 10.1111/j.1469-7610.2010.02307.x 20735793 PMC3429600

[B252] Salsano E. Pollo B. Eoli M. Giordana M. T. Finocchiaro G. (2004). Expression of MATH1, a marker of cerebellar granule cell progenitors, identifies different medulloblastoma sub-types. *Neurosci. Lett.* 370 180–185. 10.1016/j.neulet.2004.08.053 15488319

[B253] Santoni M. Burattini L. Nabissi M. Morelli M. B. Berardi R. Santoni G. (2013). Essential role of Gli proteins in glioblastoma multiforme. *Curr. Protein Pept. Sci.* 14 133–140. 10.2174/1389203711314020005 23544423

[B254] Schlierf B. Friedrich R. P. Roerig P. Felsberg J. Reifenberger G. Wegner M. (2007). Expression of SoxE and SoxD genes in human gliomas. *Neuropathol. Appl. Neurobiol.* 33 621–630. 10.1111/j.1365-2990.2007.00881.x 17961134

[B255] Schuller U. Heine V. M. Mao J. Kho A. T. Dillon A. K. Han Y. G. (2008). Acquisition of granule neuron precursor identity is a critical determinant of progenitor cell competence to form Shh-induced medulloblastoma. *Cancer Cell* 14 123–134. 10.1016/j.ccr.2008.07.005 18691547 PMC2597270

[B256] Schwartzentruber J. Korshunov A. Liu X. Y. Jones D. T. Pfaff E. Jacob K. (2012). Driver mutations in histone H3.3 and chromatin remodelling genes in paediatric glioblastoma. *Nature* 482 226–231. 22286061 10.1038/nature10833

[B257] See S. J. Levin V. A. Yung W. K. Hess K. R. Groves M. D. (2004). 13-cis-retinoic acid in the treatment of recurrent glioblastoma multiforme. *Neuro Oncol.* 6 253–258. 10.1215/s1152851703000607 15279718 PMC1871997

[B258] Seeger M. Tear G. Ferres-Marco D. Goodman C. S. (1993). Mutations affecting growth cone guidance in Drosophila: genes necessary for guidance toward or away from the midline. *Neuron* 10 409–426. 10.1016/0896-6273(93)90330-t 8461134

[B259] Selvaraj P. Huang J. S. Chen A. Skalka N. Rosin-Arbesfeld R. Loh Y. P. (2015). Neurotrophic factor-alpha1 modulates NGF-induced neurite outgrowth through interaction with Wnt-3a and Wnt-5a in PC12 cells and cortical neurons. *Mol. Cell. Neurosci.* 68 222–233. 10.1016/j.mcn.2015.08.005 26276171

[B260] Shahi M. H. Afzal M. Sinha S. Eberhart C. G. Rey J. A. Fan X. (2010). Regulation of sonic hedgehog-GLI1 downstream target genes PTCH1, Cyclin D2, Plakoglobin, PAX6 and NKX2.2 and their epigenetic status in medulloblastoma and astrocytoma. *BMC Cancer* 10:614. 10.1186/1471-2407-10-614 PMC2989332 21059263

[B261] Shahi M. H. Lorente A. Castresana J. S. (2008). Hedgehog signalling in medulloblastoma, glioblastoma and neuroblastoma. *Oncol. Rep.* 19 681–688. 18288402

[B262] Shevchenko V. Arnotskaya N. Korneyko M. Zaytsev S. Khotimchenko Y. Sharma H. (2019). Proteins of the Wnt signaling pathway as targets for the regulation of CD133+ cancer stem cells in glioblastoma. *Oncol. Rep.* 41 3080–3088. 30864699 10.3892/or.2019.7043

[B263] Shi Y. He B. You L. Jablons D. M. (2007). Roles of secreted frizzled-related proteins in cancer. *Acta Pharmacol. Sin.* 28 1499–1504. 10.1111/j.1745-7254.2007.00692.x 17723183

[B264] Shu T. Butz K. G. Plachez C. Gronostajski R. M. Richards L. J. (2003). Abnormal development of forebrain midline glia and commissural projections in Nfia knock-out mice. *J. Neurosci.* 23 203–212. 10.1523/jneurosci.23-01-00203.2003 12514217 PMC6742120

[B265] Singh S. K. Clarke I. D. Terasaki M. Bonn V. E. Hawkins C. Squire J. (2003). Identification of a cancer stem cell in human brain tumors. *Cancer Res.* 63 5821–5828. 14522905

[B266] Singh S. K. Fiorelli R. Kupp R. Rajan S. Szeto E. Lo Cascio C. (2016). Post-translational modifications of OLIG2 regulate glioma invasion through the TGF-beta pathway. *Cell Rep.* 16 950–966. 10.1016/j.celrep.2016.06.045 27396340 PMC4963280

[B267] Singh S. K. Hawkins C. Clarke I. D. Squire J. A. Bayani J. Hide T. (2004). Identification of human brain tumour initiating cells. *Nature* 432 396–401. 15549107 10.1038/nature03128

[B268] Slamon D. J. Cline M. J. (1984). Expression of cellular oncogenes during embryonic and fetal development of the mouse. *Proc. Natl. Acad. Sci. USA* 81 7141–7145. 10.1073/pnas.81.22.7141 6594688 PMC392093

[B269] Smits A. Funa K. (1998). Platelet-derived growth factor (PDGF) in primary brain tumours of neuroglial origin. *Histol. Histopathol.* 13 511–520. 9589905 10.14670/HH-13.511

[B270] Soltysova A. Altanerova V. Altaner C. (2005). Cancer stem cells. *Neoplasma* 52 435–440. 16284686

[B271] Somasundaram K. Reddy S. P. Vinnakota K. Britto R. Subbarayan M. Nambiar S. (2005). Upregulation of ASCL1 and inhibition of Notch signaling pathway characterize progressive astrocytoma. *Oncogene* 24 7073–7083. 10.1038/sj.onc.1208865 16103883

[B272] Son M. J. Woolard K. Nam D. H. Lee J. Fine H. A. (2009). SSEA-1 is an enrichment marker for tumor-initiating cells in human glioblastoma. *Cell Stem Cell* 4 440–452. 10.1016/j.stem.2009.03.003 19427293 PMC7227614

[B273] Song H. R. Gonzalez-Gomez I. Suh G. S. Commins D. L. Sposto R. Gilles F. H. (2010). Nuclear factor IA is expressed in astrocytomas and is associated with improved survival. *Neuro Oncol.* 12 122–132. 10.1093/neuonc/nop044 20150379 PMC2940580

[B274] Steele-Perkins G. Plachez C. Butz K. G. Yang G. Bachurski C. J. Kinsman S. L. (2005). The transcription factor gene Nfib is essential for both lung maturation and brain development. *Mol. Cell. Biol.* 25 685–698. 10.1128/mcb.25.2.685-698.2005 15632069 PMC543431

[B275] Stiles C. D. Rowitch D. H. (2008). Glioma stem cells: a midterm exam. *Neuron* 58 832–846. 10.1016/j.neuron.2008.05.031 18579075

[B276] Stockhausen M. T. Kristoffersen K. Poulsen H. S. (2010). The functional role of Notch signaling in human gliomas. *Neuro Oncol.* 12 199–211. 10.1093/neuonc/nop022 20150387 PMC2940575

[B277] Stolt C. C. Lommes P. Friedrich R. P. Wegner M. (2004). Transcription factors Sox8 and Sox10 perform non-equivalent roles during oligodendrocyte development despite functional redundancy. *Development* 131 2349–2358. 10.1242/dev.01114 15102707

[B278] Stolt C. C. Lommes P. Sock E. Chaboissier M. C. Schedl A. Wegner M. (2003). The Sox9 transcription factor determines glial fate choice in the developing spinal cord. *Genes Dev.* 17 1677–1689. 10.1101/gad.259003 12842915 PMC196138

[B279] Stolt C. C. Rehberg S. Ader M. Lommes P. Riethmacher D. Schachner M. (2002). Terminal differentiation of myelin-forming oligodendrocytes depends on the transcription factor Sox10. *Genes Dev.* 16 165–170. 10.1101/gad.215802 11799060 PMC155320

[B280] Stolt C. C. Schlierf A. Lommes P. Hillgartner S. Werner T. Kosian T. (2006). SoxD proteins influence multiple stages of oligodendrocyte development and modulate SoxE protein function. *Dev. Cell* 11 697–709. 10.1016/j.devcel.2006.08.011 17084361

[B281] Stolt C. C. Schmitt S. Lommes P. Sock E. Wegner M. (2005). Impact of transcription factor Sox8 on oligodendrocyte specification in the mouse embryonic spinal cord. *Dev. Biol.* 281 309–317. 10.1016/j.ydbio.2005.03.010 15893981

[B282] Streit A. Lee K. J. Woo I. Roberts C. Jessell T. M. Stern C. D. (1998). Chordin regulates primitive streak development and the stability of induced neural cells, but is not sufficient for neural induction in the chick embryo. *Development* 125 507–519. 9425145 10.1242/dev.125.3.507

[B283] Stringer B. W. Bunt J. Day B. W. Barry G. Jamieson P. R. Ensbey K. S. (2016). Nuclear factor one B (NFIB) encodes a subtype-specific tumour suppressor in glioblastoma. *Oncotarget* 7 29306–29320. 10.18632/oncotarget.8720 27083054 PMC5045397

[B284] Stuart E. T. Haffner R. Oren M. Gruss P. (1995). Loss of p53 function through PAX-mediated transcriptional repression. *EMBO J.* 14 5638–5645. 10.1002/j.1460-2075.1995.tb00251.x 8521821 PMC394679

[B285] Sturm D. Witt H. Hovestadt V. Khuong-Quang D. A. Jones D. T. Konermann C. (2012). Hotspot mutations in H3F3A and IDH1 define distinct epigenetic and biological subgroups of glioblastoma. *Cancer Cell* 22 425–437. 23079654 10.1016/j.ccr.2012.08.024

[B286] Su X. Liu X. Ni L. Shi W. Zhu H. Shi J. (2016). GFAP expression is regulated by Pax3 in brain glioma stem cells. *Oncol. Rep.* 36 1277–1284. 10.3892/or.2016.4917 27432276

[B287] Sun M. Thomas M. J. Herder R. Bofenkamp M. L. Selleck S. B. O’connor M. B. (2007). Presynaptic contributions of chordin to hippocampal plasticity and spatial learning. *J. Neurosci.* 27 7740–7750. 10.1523/jneurosci.1604-07.2007 17634368 PMC6672865

[B288] Sun Y. Meijer D. H. Alberta J. A. Mehta S. Kane M. F. Tien A. C. (2011). Phosphorylation state of Olig2 regulates proliferation of neural progenitors. *Neuron* 69 906–917. 10.1016/j.neuron.2011.02.005 21382551 PMC3065213

[B289] Sun Y. Zhang W. Chen D. Lv Y. Zheng J. Lilljebjorn H. (2014). A glioma classification scheme based on coexpression modules of EGFR and PDGFRA. *Proc. Natl. Acad. Sci. U.S.A.* 111 3538–3543. 10.1073/pnas.1313814111 24550449 PMC3948229

[B290] Sutter R. Shakhova O. Bhagat H. Behesti H. Sutter C. Penkar S. (2010). Cerebellar stem cells act as medulloblastoma-initiating cells in a mouse model and a neural stem cell signature characterizes a subset of human medulloblastomas. *Oncogene* 29 1845–1856. 10.1038/onc.2009.472 20062081

[B291] Suva M. L. Tirosh I. (2020). The glioma stem cell model in the era of single-cell genomics. *Cancer Cell* 37 630–636. 10.1016/j.ccell.2020.04.001 32396858

[B292] Suva M. L. Rheinbay E. Gillespie S. M. Patel A. P. Wakimoto H. Rabkin S. D. (2014). Reconstructing and reprogramming the tumor-propagating potential of glioblastoma stem-like cells. *Cell* 157 580–594. 10.1016/j.cell.2014.02.030 24726434 PMC4004670

[B293] Swartling F. J. Savov V. Persson A. I. Chen J. Hackett C. S. Northcott P. A. (2012). Distinct neural stem cell populations give rise to disparate brain tumors in response to N-MYC. *Cancer Cell* 21 601–613. 10.1016/j.ccr.2012.04.012 22624711 PMC3360885

[B294] Taipale J. Chen J. K. Cooper M. K. Wang B. Mann R. K. Milenkovic L. (2000). Effects of oncogenic mutations in smoothened and patched can be reversed by cyclopamine. *Nature* 406 1005–1009. 10.1038/35023008 10984056

[B295] Takebayashi H. Nabeshima Y. Yoshida S. Chisaka O. Ikenaka K. Nabeshima Y. (2002). The basic helix-loop-helix factor olig2 is essential for the development of motoneuron and oligodendrocyte lineages. *Curr. Biol.* 12 1157–1163. 10.1016/s0960-9822(02)00926-0 12121626

[B296] Taylor M. D. Liu L. Raffel C. Hui C. C. Mainprize T. G. Zhang X. (2002). Mutations in SUFU predispose to medulloblastoma. *Nat. Genet.* 31 306–310. 10.1038/ng916 12068298

[B297] Tchougounova E. Jiang Y. Brasater D. Lindberg N. Kastemar M. Asplund A. (2009). Sox5 can suppress platelet-derived growth factor B-induced glioma development in Ink4a-deficient mice through induction of acute cellular senescence. *Oncogene* 28 1537–1548. 10.1038/onc.2009.9 19219070

[B298] Thompson M. C. Fuller C. Hogg T. L. Dalton J. Finkelstein D. Lau C. C. (2006). Genomics identifies medulloblastoma subgroups that are enriched for specific genetic alterations. *J. Clin. Oncol.* 24 1924–1931. 10.1200/jco.2005.04.4974 16567768

[B299] Tirosh I. Izar B. Prakadan S. M. Wadsworth M. H. Treacy D. Trombetta J. J. (2016). Dissecting the multicellular ecosystem of metastatic melanoma by single-cell RNA-seq. *Science* 352 189–196. 27124452 10.1126/science.aad0501 PMC4944528

[B300] Tsigelny I. F. Mukthavaram R. Kouznetsova V. L. Chao Y. Babic I. Nurmemmedov E. (2017). Multiple spatially related pharmacophores define small molecule inhibitors of OLIG2 in glioblastoma. *Oncotarget* 8 22370–22384. 10.18632/oncotarget.5633 26517684 PMC5410230

[B301] Tso J. L. Yang S. Menjivar J. C. Yamada K. Zhang Y. Hong I. (2015). Bone morphogenetic protein 7 sensitizes O6-methylguanine methyltransferase expressing-glioblastoma stem cells to clinically relevant dose of temozolomide. *Mol. Cancer* 14:189. 10.1186/s12943-015-0459-1 PMC4636799 26546412

[B302] Uchida N. Buck D. W. He D. Reitsma M. J. Masek M. Phan T. V. (2000). Direct isolation of human central nervous system stem cells. *Proc. Natl. Acad. Sci. U.S.A.* 97 14720–14725. 11121071 10.1073/pnas.97.26.14720 PMC18985

[B303] Ueda R. Iizuka Y. Yoshida K. Kawase T. Kawakami Y. Toda M. (2004). Identification of a human glioma antigen, SOX6, recognized by patients’ sera. *Oncogene* 23 1420–1427. 10.1038/sj.onc.1207252 14691456

[B304] Ueda R. Kinoshita E. Ito R. Kawase T. Kawakami Y. Toda M. (2008). Induction of protective and therapeutic antitumor immunity by a DNA vaccine with a glioma antigen, SOX6. *Int. J. Cancer* 122 2274–2279. 10.1002/ijc.23366 18224680

[B305] Vanner R. J. Remke M. Gallo M. Selvadurai H. J. Coutinho F. Lee L. (2014). Quiescent sox2(+) cells drive hierarchical growth and relapse in sonic hedgehog subgroup medulloblastoma. *Cancer Cell* 26 33–47. 10.1016/j.ccr.2014.05.005 24954133 PMC4441014

[B306] Venteicher A. S. Tirosh I. Hebert C. Yizhak K. Neftel C. Filbin M. G. (2017). Decoupling genetics, lineages, and microenvironment in IDH-mutant gliomas by single-cell RNA-seq. *Science* 355:eaai8478. 10.1126/science.aai8478 28360267 PMC5519096

[B307] Verhaak R. G. Hoadley K. A. Purdom E. Wang V. Qi Y. Wilkerson M. D. (2010). Integrated genomic analysis identifies clinically relevant subtypes of glioblastoma characterized by abnormalities in PDGFRA, IDH1, EGFR, and NF1. *Cancer Cell* 17 98–110. 10.1016/j.ccr.2009.12.020 20129251 PMC2818769

[B308] Videla Richardson G. A. Garcia C. P. Roisman A. Slavutsky I. Fernandez Espinosa D. D. Romorini L. (2016). Specific preferences in lineage choice and phenotypic plasticity of glioma stem cells under BMP4 and noggin influence. *Brain Pathol.* 26 43–61. 10.1111/bpa.12263 25808628 PMC8029422

[B309] Voumvourakis K. I. Antonelou R. Kitsos D. K. Stamboulis E. Tsiodras S. (2011). TGF-beta/BMPs: crucial crossroad in neural autoimmune disorders. *Neurochem. Int.* 59 542–550. 10.1016/j.neuint.2011.06.004 21718734

[B310] Vue T. Y. Kim E. J. Parras C. M. Guillemot F. Johnson J. E. (2014). Ascl1 controls the number and distribution of astrocytes and oligodendrocytes in the gray matter and white matter of the spinal cord. *Development* 141 3721–3731. 10.1242/dev.105270 25249462 PMC4197573

[B311] Vue T. Y. Kollipara R. K. Borromeo M. D. Smith T. Mashimo T. Burns D. K. (2020). ASCL1 regulates neurodevelopmental transcription factors and cell cycle genes in brain tumors of glioma mouse models. *Glia* 68 2613–2630. 10.1002/glia.23873 32573857 PMC7587013

[B312] Wang K. H. Brose K. Arnott D. Kidd T. Goodman C. S. Henzel W. (1999). Biochemical purification of a mammalian slit protein as a positive regulator of sensory axon elongation and branching. *Cell* 96 771–784. 10.1016/s0092-8674(00)80588-7 10102266

[B313] Wang L. He S. Yuan J. Mao X. Cao Y. Zong J. (2012). Oncogenic role of SOX9 expression in human malignant glioma. *Med. Oncol.* 29 3484–3490. 10.1007/s12032-012-0267-z 22714060

[B314] Wang Q. Fang W. H. Krupinski J. Kumar S. Slevin M. Kumar P. (2008). Pax genes in embryogenesis and oncogenesis. *J. Cell Mol. Med.* 12 2281–2294. 18627422 10.1111/j.1582-4934.2008.00427.x PMC4514106

[B315] Wang Y. Lin L. Lai H. Parada L. F. Lei L. (2013). Transcription factor Sox11 is essential for both embryonic and adult neurogenesis. *Dev. Dyn.* 242 638–653. 10.1002/dvdy.23962 23483698

[B316] Ward R. J. Lee L. Graham K. Satkunendran T. Yoshikawa K. Ling E. (2009). Multipotent CD15+ cancer stem cells in patched-1-deficient mouse medulloblastoma. *Cancer Res.* 69 4682–4690. 10.1158/0008-5472.can-09-0342 19487286

[B317] Watabe T. Miyazono K. (2009). Roles of TGF-beta family signaling in stem cell renewal and differentiation. *Cell Res.* 19 103–115. 10.1038/cr.2008.323 19114993

[B318] Watanabe T. Nobusawa S. Kleihues P. Ohgaki H. (2009). IDH1 mutations are early events in the development of astrocytomas and oligodendrogliomas. *Am. J. Pathol.* 174 1149–1153. 10.2353/ajpath.2009.080958 19246647 PMC2671348

[B319] Wegner M. Stolt C. C. (2005). From stem cells to neurons and glia: a Soxist’s view of neural development. *Trends Neurosci.* 28 583–588. 10.1016/j.tins.2005.08.008 16139372

[B320] Wu A. Aldape K. Lang F. F. (2010). High rate of deletion of chromosomes 1p and 19q in insular oligodendroglial tumors. *J. Neurooncol.* 99 57–64. 10.1007/s11060-009-0100-5 20035368 PMC2891585

[B321] Wu G. Broniscer A. Mceachron T. A. Lu C. Paugh B. S. Becksfort J. (2012). Somatic histone H3 alterations in pediatric diffuse intrinsic pontine gliomas and non-brainstem glioblastomas. *Nat. Genet.* 44 251–253. 10.1038/ng.1102 22286216 PMC3288377

[B322] Wu W. Wong K. Chen J. Jiang Z. Dupuis S. Wu J. Y. (1999). Directional guidance of neuronal migration in the olfactory system by the protein Slit. *Nature* 400 331–336. 10.1038/22477 10432110 PMC2041931

[B323] Xing Z. Y. Sun L. G. Guo W. J. (2015). Elevated expression of Notch-1 and EGFR induced apoptosis in glioblastoma multiforme patients. *Clin. Neurol. Neurosurg.* 131 54–58. 10.1016/j.clineuro.2015.01.018 25704190

[B324] Xu Q. Yuan X. Liu G. Black K. L. Yu J. S. (2008). Hedgehog signaling regulates brain tumor-initiating cell proliferation and portends shorter survival for patients with PTEN-coexpressing glioblastomas. *Stem Cells* 26 3018–3026. 10.1634/stemcells.2008-0459 18787206

[B325] Yamaguchi T. P. (2001). Heads or tails: wnts and anterior-posterior patterning. *Curr. Biol.* 11 R713–R724. 11553348 10.1016/s0960-9822(01)00417-1

[B326] Yang Z. J. Ellis T. Markant S. L. Read T. A. Kessler J. D. Bourboulas M. (2008). Medulloblastoma can be initiated by deletion of patched in lineage-restricted progenitors or stem cells. *Cancer Cell* 14 135–145. 10.1016/j.ccr.2008.07.003 18691548 PMC2538687

[B327] Yiin J. J. Hu B. Jarzynka M. J. Feng H. Liu K. W. Wu J. Y. (2009). Slit2 inhibits glioma cell invasion in the brain by suppression of Cdc42 activity. *Neuro Oncol.* 11 779–789. 10.1215/15228517-2009-017 20008733 PMC2802398

[B328] Yoon K. Gaiano N. (2005). Notch signaling in the mammalian central nervous system: insights from mouse mutants. *Nat. Neurosci.* 8 709–715. 10.1038/nn1475 15917835

[B329] Ypsilanti A. R. Zagar Y. Chedotal A. (2010). Moving away from the midline: new developments for Slit and Robo. *Development* 137 1939–1952. 10.1242/dev.044511 20501589

[B330] Yu K. Lin C. J. Hatcher A. Lozzi B. Kong K. Huang-Hobbs E. (2020). PIK3CA variants selectively initiate brain hyperactivity during gliomagenesis. *Nature* 578 166–171. 10.1038/s41586-020-1952-2 31996845 PMC7577741

[B331] Yu Z. Pestell T. G. Lisanti M. P. Pestell R. G. (2012). Cancer stem cells. *Int. J. Biochem. Cell Biol.* 44 2144–2151. 22981632 10.1016/j.biocel.2012.08.022 PMC3496019

[B332] Zbinden M. Duquet A. Lorente-Trigos A. Ngwabyt S. N. Borges I. Ruiz I. (2010). NANOG regulates glioma stem cells and is essential in vivo acting in a cross-functional network with GLI1 and p53. *EMBO J.* 29 2659–2674. 10.1038/emboj.2010.137 20581802 PMC2928692

[B333] Zeng H. Yang Z. Xu N. Liu B. Fu Z. Lian C. (2017). Connective tissue growth factor promotes temozolomide resistance in glioblastoma through TGF-beta1-dependent activation of Smad/ERK signaling. *Cell Death Dis.* 8:e2885. 10.1038/cddis.2017.248 28617438 PMC5520906

[B334] Zhang H. Geng D. Gao J. Qi Y. Shi Y. Wang Y. (2016). Expression and significance of Hippo/YAP signaling in glioma progression. *Tumour Biol.* 10.1007/s13277-016-5318-1 Online ahead of print. 27718125

[B335] Zhang J. Jiang H. Shao J. Mao R. Liu J. Ma Y. (2014). SOX4 inhibits GBM cell growth and induces G0/G1 cell cycle arrest through Akt-p53 axis. *BMC Neurol.* 14:207. 10.1186/s12883-014-0207-y PMC4233052 25366337

[B336] Zhang L. He X. Liu X. Zhang F. Huang L. F. Potter A. S. (2019). Single-Cell transcriptomics in medulloblastoma reveals tumor-initiating progenitors and oncogenic cascades during tumorigenesis and relapse. *Cancer Cell* 36 302–318 e307. 31474569 10.1016/j.ccell.2019.07.009 PMC6760242

[B337] Zhang M. Yang D. Gold B. (2019). Origin of mutations in genes associated with human glioblastoma multiform cancer: random polymerase errors versus deamination. *Heliyon* 5:e01265. 10.1016/j.heliyon.2019.e01265 30899826 PMC6407082

[B338] Zhang N. Wei P. Gong A. Chiu W. T. Lee H. T. Colman H. (2011). FoxM1 promotes beta-catenin nuclear localization and controls Wnt target-gene expression and glioma tumorigenesis. *Cancer Cell* 20 427–442. 10.1016/j.ccr.2011.08.016 22014570 PMC3199318

[B339] Zhang X. P. Zheng G. Zou L. Liu H. L. Hou L. H. Zhou P. (2008). Notch activation promotes cell proliferation and the formation of neural stem cell-like colonies in human glioma cells. *Mol. Cell. Biochem.* 307 101–108. 10.1007/s11010-007-9589-0 17849174

[B340] Zheng H. Ying H. Wiedemeyer R. Yan H. Quayle S. N. Ivanova E. V. (2010). PLAGL2 regulates wnt signaling to impede differentiation in neural stem cells and gliomas. *Cancer Cell* 17 497–509. 10.1016/j.ccr.2010.03.020 20478531 PMC2900858

[B341] Zhou Q. Anderson D. J. (2002). The bHLH transcription factors OLIG2 and OLIG1 couple neuronal and glial subtype specification. *Cell* 109 61–73. 10.1016/s0092-8674(02)00677-3 11955447

[B342] Zhu X. Zuo H. Maher B. J. Serwanski D. R. Loturco J. J. Lu Q. R. (2012). Olig2-dependent developmental fate switch of NG2 cells. *Development* 139 2299–2307. 10.1242/dev.078873 22627280 PMC3367441

[B343] Zong H. Parada L. F. Baker S. J. (2015). Cell of origin for malignant gliomas and its implication in therapeutic development. *Cold Spring Harb. Perspect. Biol.* 7:a020610. 10.1101/cshperspect.a020610 25635044 PMC4448618

[B344] Zuccarini M. Giuliani P. Ziberi S. Carluccio M. Iorio P. D. Caciagli F. (2018). The role of wnt signal in glioblastoma development and progression: a possible new pharmacological target for the therapy of this tumor. *Genes* 9:105. 10.3390/genes9020105 29462960 PMC5852601

